# Regulation of ADAMTS Proteases

**DOI:** 10.3389/fmolb.2021.701959

**Published:** 2021-06-29

**Authors:** Keron W. J. Rose, Nandaraj Taye, Stylianos Z. Karoulias, Dirk Hubmacher

**Affiliations:** Orthopaedic Research Laboratories, Leni and Peter W. May Department of Orthopaedics, Icahn School of Medicine at Mount Sinai, New York, NY, United States

**Keywords:** extracellular matrix, arthritis, small molecule inhibitor, posttranslational modifications, alternative splicing, cartilage, aggrecan

## Abstract

A disintegrin and metalloprotease with thrombospondin type I motifs (ADAMTS) proteases are secreted metalloproteinases that play key roles in the formation, homeostasis and remodeling of the extracellular matrix (ECM). The substrate spectrum of ADAMTS proteases can range from individual ECM proteins to entire families of ECM proteins, such as the hyalectans. ADAMTS-mediated substrate cleavage is required for the formation, remodeling and physiological adaptation of the ECM to the needs of individual tissues and organ systems. However, ADAMTS proteases can also be involved in the destruction of tissues, resulting in pathologies such as arthritis. Specifically, ADAMTS4 and ADAMTS5 contribute to irreparable cartilage erosion by degrading aggrecan, which is a major constituent of cartilage. Arthritic joint damage is a major contributor to musculoskeletal morbidity and the most frequent clinical indication for total joint arthroplasty. Due to the high sequence homology of ADAMTS proteases in their catalytically active site, it remains a formidable challenge to design ADAMTS isotype-specific inhibitors that selectively inhibit ADAMTS proteases responsible for tissue destruction without affecting the beneficial functions of other ADAMTS proteases. In vivo, proteolytic activity of ADAMTS proteases is regulated on the transcriptional and posttranslational level. Here, we review the current knowledge of mechanisms that regulate ADAMTS protease activity in tissues including factors that induce ADAMTS gene expression, consequences of posttranslational modifications such as furin processing, the role of endogenous inhibitors and pharmacological approaches to limit ADAMTS protease activity in tissues, which almost exclusively focus on inhibiting the aggrecanase activity of ADAMTS4 and ADAMTS5.

## Introduction

The a disintegrin and metalloprotease with thrombospondin type I motifs (ADAMTS) protease family comprises 19 secreted metalloproteases with a broad substrate and functional spectrum that is ever expanding due to recent advances in mass spectrometry-based substrate identification and due to the characterization of knock-out mouse models for most of the ADAMTS proteases (Puente et al., 2003; Kleifeld et al., 2011; Dubail and Apte, 2015; Savickas and Auf dem Keller, 2017; Apte, 2020; Satz-Jacobowitz and Hubmacher, 2021). Commensurate with the broad substrate and functional spectrum, ADAMTS proteases play major roles in organ development and tissue homeostasis by regulating extracellular matrix (ECM) formation, remodeling and homeostatic adaptation. Well characterized examples include the promotion of collagen fibrillogenesis by ADAMTS2, which cleaves the N-terminal propeptide of procollagen, or the remodeling of proteoglycan-rich ECMs by ADAMTS5, 9 and 20 during interdigital web regression and palate closure (Colige et al., 1999; Le Goff et al., 2006; McCulloch et al., 2009; Enomoto et al., 2010; Dubail et al., 2014). On the other hand, ADAMTS proteases are also involved in the pathogenesis of acquired and congenital connective tissue disorders, most prominently in arthritis, where ADAMTS4 and ADAMTS5 degrade aggrecan and contribute to the erosion of articular cartilage and joint degeneration (Glasson et al., 2005; Ilic et al., 2007; Song et al., 2007; Verma and Dalal, 2011; Santamaria, 2020). Examples of inherited connective tissue disorders that are caused by mutations in ADAMTS proteases, which likely reduce protease activity in the ECM, include Weill-Marchesani syndrome (*ADAMTS10*, *ADAMTS17*), dermatosparaxis Ehlers Danlos syndrome (*ADAMTS2*), isolated heart valve disease (*ADAMTS19*), or congenital thrombotic thrombocytopenic purpura (*ADAMTS13*) (Colige et al., 1999; Dagoneau et al., 2004; Sadler, 2008; Morales et al., 2009; Evans et al., 2020; Karoulias et al., 2020; Wunnemann et al., 2020).

**FIGURE 1:**
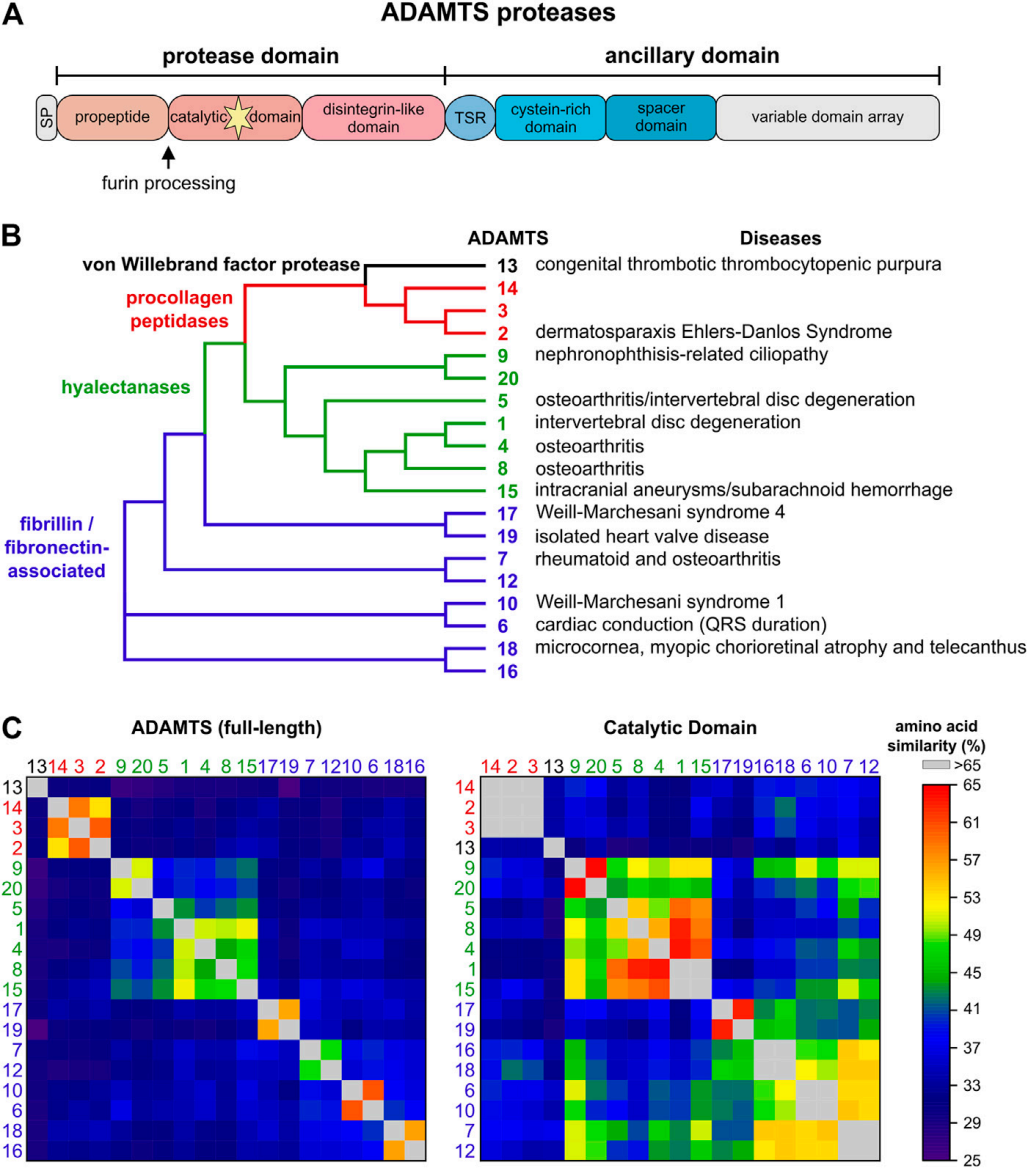
The human ADAMTS protease family. **(A)** Domain organization of ADAMTS proteases. ADAMTS protease show identical domain organization of the protease domain and parts of the ancillary domain. The C-terminal variable domain arrays include between 0 (ADAMTS4) - 14 (ADAMTS9, ADAMTS20) thrombospondin type I motif (TSR) domains interspersed with additional domains unique to ADAMTS protease pairs, such as GON1, PLAC or CUB domains. **(B)** Phylogenetic tree of the human ADAMTS proteases generated with Clustal Omega using the full-length protein sequences of the ADAMTS protease (Madeira et al., 2019). Four ADAMTS subfamilies are evident: ADAMTS13 (black), the procollagen peptidases (red), the hyalectanases (green), and ADAMTS proteases associated with cleavage and/or binding to fibrillin and/or fibronectin (blue). The latter subfamily consists of four distinct pairs of ADAMTS proteases. Disorders associated with individual ADAMTS proteases are indicated on the right. **(C)** Heat map showing the amino acid similarities of full-length ADAMTS proteases (left) and the respective catalytic domains (right). The full-length ADAMTS proteases cluster in the same groups as indicated in A with little similarities to proteases outside of these groups. These clusters are mainly defined by the ancillary domain and the propeptide domain. However, a similar analysis of the catalytic domain shows that amino acid similarity still separates ADAMTS13 and the procollagen peptidases but that the boundaries that separated the hyalectanases and the fibrillin/fibronectin associated ADAMTS protease are now less well defined. This underscores the challenge of generating specific inhibitors for individual ADAMTS proteases by targeting the catalytic domain.

ADAMTS proteases can be divided into four groups based on their substrate spectrum: ADAMTS13, procollagen peptidases, hyalectanases and ADAMTS proteases associated with fibrillin and fibronectin (Figures 1A,B). This is reflected in the phylogenetic tree based on the amino acid similarity of the 19 human ADAMTS proteases (Figure 1B). The grouping of ADAMTS proteases is mainly driven by their ancillary domains, since a similar analysis with the catalytic domain shows much higher amino acid conservation and the identity of the hyalectanases and the fibrillin/fibronectin-associated ADAMTS proteases is almost lost (Figure 1C). The individual ADAMTS subgroups were generated likely by gene duplication events during vertebrate evolution and originate from six *ADAMTS* genes that were identified in the basic chordate *Ciona intestinalis* (Huxley-Jones et al., 2005). The fibrillin/fibronectin-associated ADAMTS proteases are subdivided into four pairs and *ADAMTS*13, 17, and 19 do not have orthologues in *C. intestinalis*, which suggests that these *ADAMTS* proteases evolved in vertebrates (Huxley-Jones et al., 2005). The phylogenetic tree also indicates that individual ADAMTS proteases have a “sister” protease, with the exception of *ADAMTS5*, 8, 13, and 14. Functional redundancy or genetic interactions were demonstrated for several of these ADAMTS pairs (McCulloch et al., 2009; Mead et al., 2018; Mead et al., 2021). The *ADAMTS9*/*ADAMTS20* pair is conserved down to the worm *Caenorhabditis elegans*, where *Gon-1* is the only ADAMTS orthologue (Blelloch et al., 1999; Blelloch and Kimble, 1999). Overall, the evolutionary expansion of the ADAMTS protease family likely reflects the evolution of the ECM, which gained tremendous structural and functional complexity when transitioning from invertebrates to vertebrates (Nicholson et al., 2005; Hynes, 2012; Brunet et al., 2015).

**FIGURE 2:**
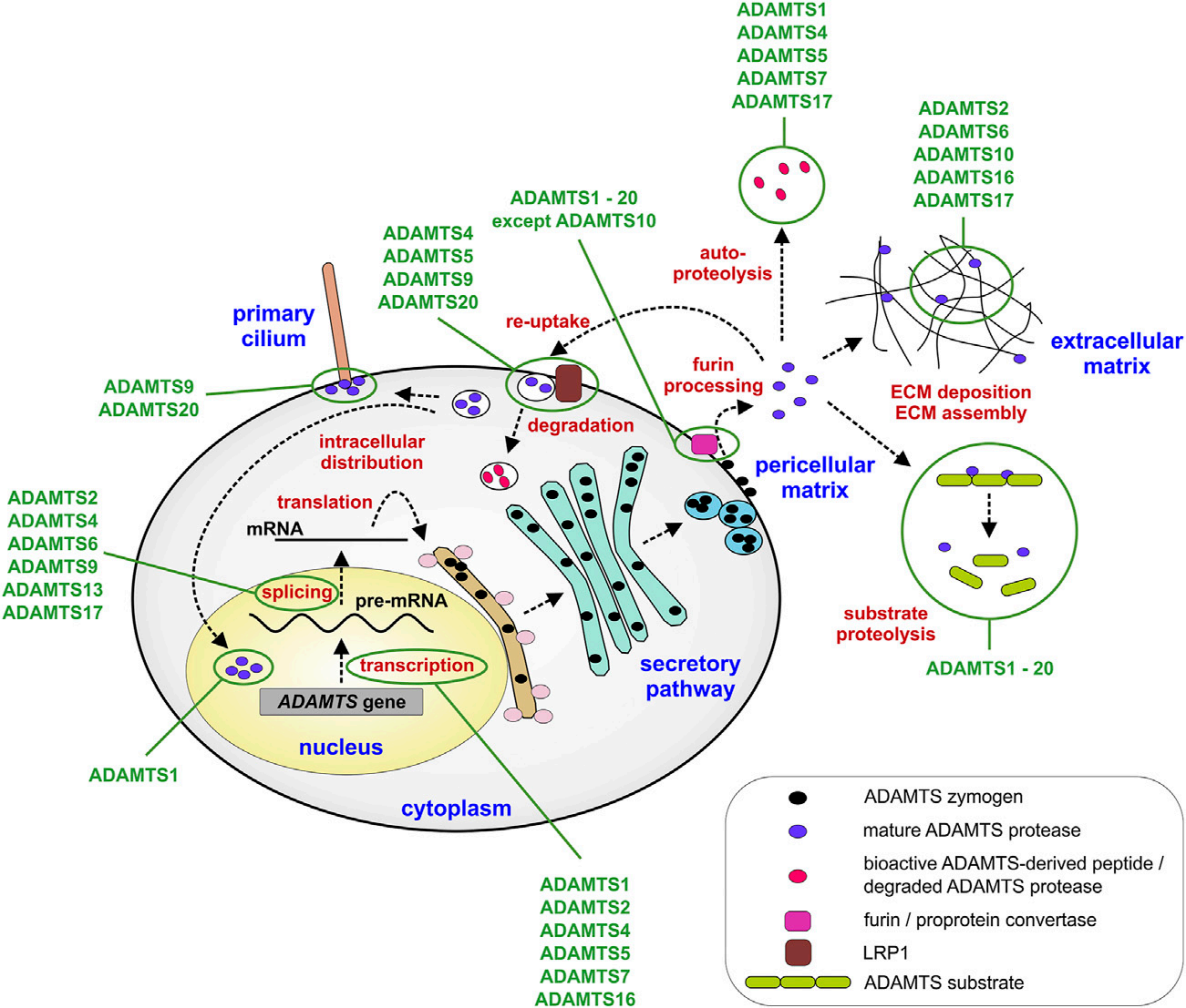
Steps that regulate ADAMTS protease expression and activity. ADAMTS proteases can be regulated transcriptionally, during mRNA splicing or translation into protein. Major posttranslational regulatory steps include furin-mediated activation of ADAMTS proteases and the localization of ADAMTS protease activity in the pericellular or extracellular matrix. In addition, re-uptake of active ADAMTS proteases mediated by LRP1 results in the localization of ADAMTS protease activity to intracellular compartments, such as the primary cilium or the nucleus or in the clearance of protease activity from the extracellular matrix. Cellular and extracellular compartments are labeled in blue and points of regulation in red. Some ADAMTS proteases discussed in the review are depicted. Exceptions, such as activation of ADAMTS proteases in the secretory pathway or the ECM or absence of furin processing are described in the text only.

While it is certainly true that most of the biology of ADAMTS proteases, or any protease for that matter, is ultimately defined by the consequences of substrate cleavage *in vivo*, it is equally important to elucidate the mechanisms by which ADAMTS protease activity is regulated during tissue development and maturation to fully understand how ADAMTS proteases work (Jones and Riley, 2005; Apte, 2020; Satz-Jacobowitz and Hubmacher, 2021). By regulating ADAMTS protease activity itself, the fate of entire groups of ECM substrates can be simultaneously altered and therefore, these proteases could be seen akin to nodes in a regulatory network that could be targeted in disorders such as arthritis, where ADAMTS protease activity is pathologically upregulated. The regulation of ADAMTS protease activity can be achieved on multiple levels, such as transcriptional regulation, posttranscriptional and posttranslational modifications or on the level of ADAMTS activation by furin processing (Figure 2). Further, regulating the access and binding of ADAMTS proteases to their substrates and ECM scaffolds can add additional layers of complexity to spatially control ADAMTS protease activity in the ECM. Finally, endogenous and exogenous inhibitors can be deployed to balance ADAMTS protease activity in tissues with the translational goal to prevent connective tissue destruction and ultimately tissue failure.

Several aspects of ADAMTS proteases have been reviewed recently, including structural considerations, evolutionary aspects, the implication of ADAMTS proteases in inherited connective tissue disorders and the importance of substrate identification in understanding ADAMTS protease biology (Nicholson et al., 2005; Brunet et al., 2015; Takeda, 2016; Mead and Apte, 2018; Apte, 2020; Satz-Jacobowitz and Hubmacher, 2021). Here, we summarize recent insights into the mechanisms that regulate ADAMTS protease activity that could potentially be harnessed to modulate ADAMTS-mediated substrate cleavage, specifically in the context of degenerative connective tissue disorders. We will also provide an overview of past and current efforts to develop ADAMTS isotype-specific inhibitors that mainly target the aggrecanases ADAMTS4 and ADAMTS5 and are currently being pursued as potential disease-modifying arthritis drugs.

## Transcriptional and Posttranscriptional Regulation of ADAMTS Proteases

*ADAMTS* mRNA abundance can be regulated on the transcriptional level and through microRNAs, which may also interfere with *ADAMTS* mRNA translation. In addition, several posttranscriptional and posttranslational mechanisms including alternative splicing and furin-mediated ADAMTS protease activation can determine tissue-specific ADAMTS isoform composition with possibly distinct proteolytic activities and/or substrates.

### Transcriptional Regulation of ADAMTS Proteases

Despite the importance of ADAMTS proteases in developmental and homeostatic processes, little is known about their transcriptional activation or repression. Due to their prominence in arthritis, the regulation of *ADAMTS4* and *ADAMTS5* gene expression by pro-inflammatory cytokines has been studied to some extent (Malfait et al., 2002; Glasson et al., 2005; Bondeson et al., 2008; Kapoor et al., 2011). For example, when human chondrocytes were exposed to oncostatin or interleukin (IL)-1β, *ADAMTS4* and matrix metalloproteinase (MMP) 13 gene expression and protease activity was upregulated (El Mabrouk et al., 2007). The IL-1β signal was transduced through a combination of ERK1/2, JAK3-STAT1/3, PI3 kinase and Akt signaling suggesting a complex, possibly chondrocyte-specific signal transduction network that resulted in the induction of ADAMTS4. Tumor necrosis factor (TNF) α and IL-6 were also able to induce *ADAMTS4* and *ADAMTS5* mRNA expression in synovial cells consistent with a regulation of these two ADAMTS proteases by pro-inflammatory cytokines (Mimata et al., 2012; Uchida et al., 2017). Signal transduction of TNFα in synovial cells involved TAK1, a MAP kinase (MAPK) that can transduce signals originating from several cytokines (Xu and Lei, 2020). IL-6 signaling was mediated by MAPK, ERK1/2 and MEK. Regulation of *ADAMTS4* and *ADAMTS5* gene expression by pro-inflammatory cytokines is not restricted to arthritis and was also described in the heart and the intervertebral disc. Increased *ADAMTS4* and *ADAMTS8* mRNA expression was observed in neonatal cardiomyocytes and cardiac fibroblasts when stimulated with TNFα and IL-1β (Vistnes et al., 2014). *ADAMTS4* and *ADAMTS5* were induced in degenerating intervertebral discs due to increased levels of TNFα and IL-1β (Zhao et al., 2011; Tian et al., 2013). Stimulation of nucleus pulposus cells, which represent the inner aggrecan-producing cells of the intervertebral disc, with TNFα and IL-1β increased mRNA levels of both aggrecanases, *ADAMTS4* and *ADAMTS5*.

Outside of inflammatory regulators there is evidence that *ADAMTS1* is regulated by progesterone and luteinizing hormone during ovulation, where it may play a role in versican remodeling in the ECM surrounding the cumulus-oocyte complex (Doyle et al., 2004). Consistent with this finding, *Adamts1* knock-out mice showed abnormal ovaries and reduced fertility (Shindo et al., 2000). *ADAMTS16* regulation was also described in the genitourinary system. Follicle-stimulating hormone and forskolin, an adenylyl cyclase activator, induced *ADAMTS16* expression in fully differentiated granulosa cells, which are part of the cumulus complex that surrounds oocytes likely through stimulation of the cAMP pathway (Gao et al., 2007). In the kidneys, *ADAMTS16* was directly regulated by the zinc-finger transcription factor WT1 (Jacobi et al., 2013). ADAMTS16 plays a role in branching morphogenesis of kidneys, possibly through proteolysis of fibronectin (Schnellmann et al., 2018). In addition, *ADAMTS16* is positively regulated by the transcription factors EGR1 and SP1 and transforming growth factor (TGF) β in chondrocyte cell lines (Surridge et al., 2009). *ADAMTS2* was upregulated in osteoblastic cell lines, such as MG63 or Saos-2 when treated with IL-6. The upregulation of *ADAMTS2* mRNA by IL-6 was mediated by the JNK pathway as treatment of Saos-2 cells with a JNK specific inhibitor suppressed IL-6 induced *ADAMTS2* mRNA expression (Alper and Kockar, 2014). *ADAMTS2* expression was also induced by glucocorticoids in macrophages (Hofer et al., 2008). Since the primary function of ADAMTS2 is the processing of the N-terminal procollagen propeptide, the functional significance for *ADAMTS2* induction in macrophages during wound repair remains to be established. Recently, additional substrates for ADAMTS2 were identified in the skin, including fibronectin, which is an important part of the provisional ECM formed after injury, and several proteins linked to inflammation (Bekhouche et al., 2016; Leduc et al., 2021). Therefore, regulation of ADAMTS2 in non-collagen producing cell types could hint to a broader substrate spectrum as previously anticipated *in vivo*. Alternatively, macrophage-derived ADAMTS2 could enhance collagen fibrillogenesis during wound healing in-trans and thus augment the capacity of collagen-producing cell types to deposit collagen fibrils, if ADAMTS2-mediated removal of the collagen propeptide is a rate-limiting step. *ADAMTS7*, a potent chondrocyte differentiation inhibitor, was directly regulated by PTHrP, where it mediated the inhibition of chondrocyte hypertrophy (Bai et al., 2009).

In several cancer cell lines, expression of ADAMTS proteases was silenced through epigenetic mechanisms (Wagstaff et al., 2011; Redondo-Garcia et al., 2021). In most instances, including for *ADAMTS1*, 5 and 8, epigenetic regulation was achieved through increased methylation of the respective promotor regions (Choi et al., 2008; Kim et al., 2011; Choi et al., 2014). *ADAMTS12* is an interesting example, since it was silenced through promoter hypermethylation in colon cancer cells but it was transcriptionally activated in the surrounding stromal cells (Moncada-Pazos et al., 2009). The authors speculated that the upregulation of *ADAMTS12* in the stroma may be a response to restrain the growing *ADAMTS12*-negative tumor. In a more recent study it was indeed shown that *ADAMTS12* depletion in a lung cancer cell line resulted in increased proliferation and invasion and that *Adamts12*-deficient mice had a 5-fold increase in lung tumor burden after urethane exposure (Rabadan et al., 2020). In contrast to malignant cell types, much less is known about the regulation of ADAMTS gene expression through chromatin alterations and epigenetic mechanisms in other cell types or tissues. In cardiac development, *ADAMTS1* is repressed by BRG1-mediated chromatin remodeling until trabecular growth is completed (Stankunas et al., 2008). Upon de-repression, i.e. induction of *ADAMTS1* and its versicanase activity, the cardiac jelly, which is permissive for trabeculation, is remodeled and degraded and trabeculation ceases. Epigenetic dysregulation of several members of the ADAMTS family was identified in placentas from preterm births compared to term birth placentas (Mani et al., 2019). Specifically, *ADAMTS12* and *ADAMTS16* displayed multiple differential methylation sites where *ADAMTS12* and *ADAMTS16* were hypo- and hypermethylated in the preterm cohort, respectively. Based on gene expression and functional data the authors concluded that both proteases may be important for trophoblast invasion and the proper anchoring of the placenta. With the current pace in the development of high throughput methodology to identify epigenetic modifications and determine tissue and cell level gene expression changes, it will be exciting to define the epigenetic regulatory networks that determine ADAMTS protease activity and to explore if these mechanisms can be harnessed to either activate desired ADAMTS proteases or to silence deleterious ADAMTS protease activity *in vivo* (Li, 2021).

Collectively, regulation of *ADAMTS* gene expression through transcriptional and epigenetic mechanisms is complicated and likely tissue and cell-type specific. Despite the complexity, it appears that a subgroup of *ADAMTS* proteases respond to pro-inflammatory cytokines while other ADAMTS proteases are regulated by sex-hormones during ovulation. It will be interesting to discover the gene-regulatory networks in which *ADAMTS* proteases participate and to elucidate how these networks are spatiotemporally regulated during normal development and homeostasis or possibly dysregulated in acquired diseases. It is worth mentioning that individual ADAMTS proteases can compensate for each other and in some instances this compensation is attributed to the induction of gene expression of the respective sister protease (McCulloch et al., 2009; Mead et al., 2018; Nandadasa et al., 2019). However, nothing is known about the signals that regulate these compensatory gene expression changes, which may involve signaling through the respective ADAMTS substrates themselves.

### Alternative Splicing and Posttranslational Modifications of ADAMTS Proteases

**FIGURE 3:**
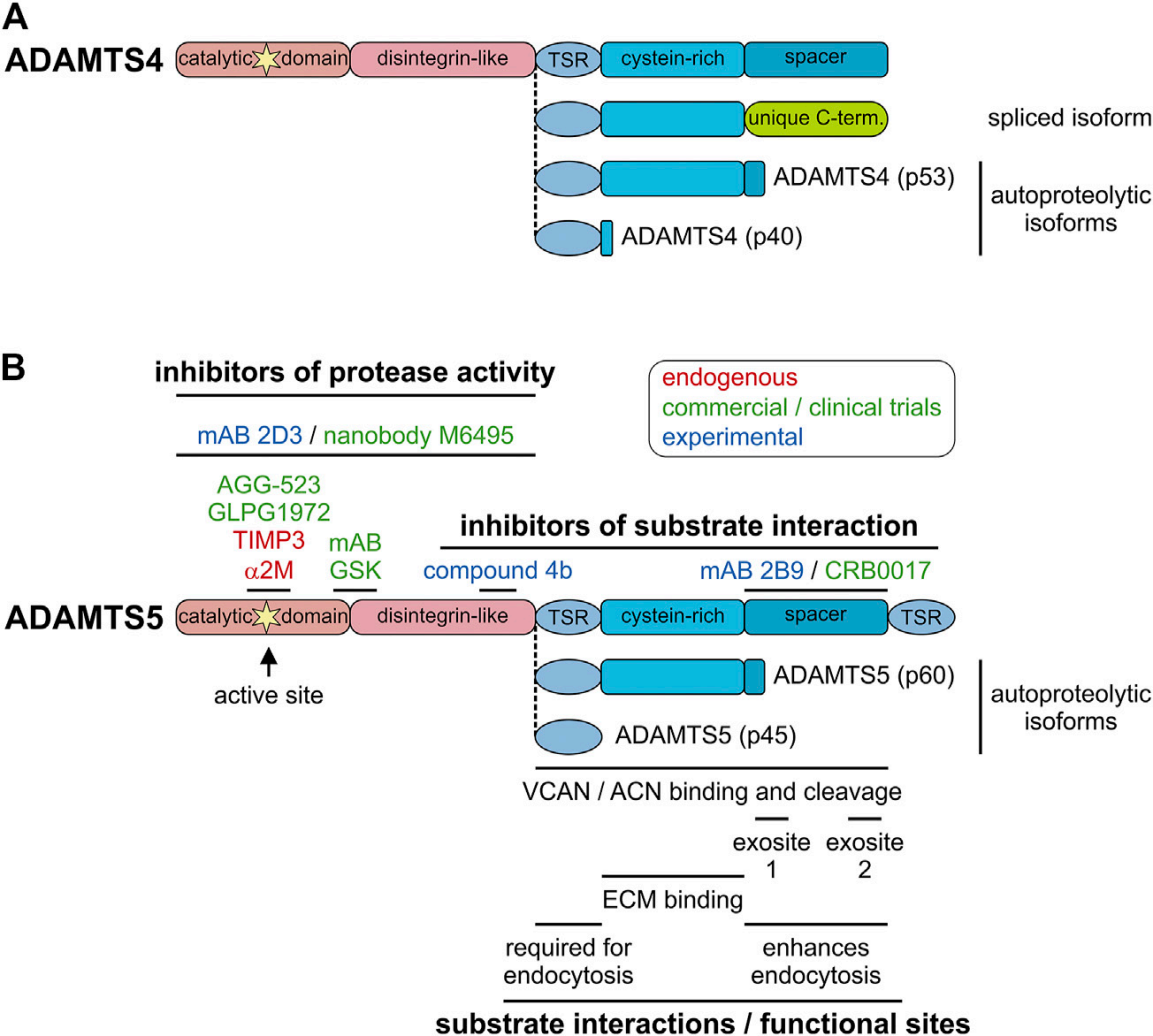
Endogenous and pharmacological inhibitors of ADAMTS proteases. **(A)** Domain organization of furin-processed mature ADAMTS4 and the organization of the ancillary domain of spliced and autoproteolytic isoforms showing the similarity to ADAMTS5 and its isoforms. **(B)** Domain organization of furin-processed mature ADAMTS5 depicting the location of endogenous and pharmacological inhibitor epitopes **(top)** and relevant functional and substrate interaction sites of ADAMTS5 **(bottom)**. The ancillary domains of the autoproteolytic isoforms p60 and p45 are depicted. Endogenous inhibitors are indicated in red, inhibitors that went through clinical trials or are in commercial product pipelines are indicated in green and experimental inhibitors reported in the literature are indicated in blue.

Alternative splicing events have the potential to augment the proteome, i.e., to increase the number of individual protein species compared to the number of protein-coding genes with predictions of up to 200,000 potentially protein-coding transcripts (Hu et al., 2015). However, for a large majority of protein isoforms, the functional role for the alternatively spliced gene products is unclear. Alternative splicing and the existence of individual *ADAMTS* isoforms was reported for *ADAMTS2*, 4, 6, 7, 9, 13, and 17, but functional differences between individual ADAMTS isoforms were only described in a few reports (Colige et al., 1999; Bevitt et al., 2003; Bevitt et al., 2005; Balic et al., 2021). A splice variant of *ADAMTS4*, which resulted in the removal of the spacer domain and the inclusion of a unique C-terminus, was detected in the synovium of arthritis patients (Figure 3A) (Wainwright et al., 2006). As a consequence, aggrecanase activity of ADAMTS4 was enhanced and it was suggested that the alternatively spliced isoform of ADAMTS4 could contribute to cartilage erosion in the arthritic joint (Hashimoto et al., 2004; Kashiwagi et al., 2004). However, targeted deletion of the spacer domain in ADAMTS4 and ADAMTS5 reduced or abolished aggrecanase and versicanase activity *in vitro* suggesting that the spacer domain is required for cleavage of aggrecan and versican (Fushimi et al., 2008; Santamaria et al., 2019). One factor which could explain these discrepancies may be the degree of contamination with heparin during ADAMTS4 preparations. Heparin specifically inhibited the aggrecanase activity of full-length ADAMTS4 and may have resulted in an underestimation of its aggrecanase activity, compared to the activity of alternatively spliced ADAMTS4 (Fushimi et al., 2008). Alternatively, the ADAMTS4 isoform without the spacer domain may have lost its capability to be sequestered to the cell-surface and as a consequence was released into the ECM/synovium, where the spacer-less ADAMTS4 isoform now could reach its substrate aggrecan (Gao et al., 2004). The alternative splicing events reported for *ADAMTS17* also occurred in the spacer domain and resulted not in the removal of the entire domain but in the deletion and insertion of shorter peptides that altered the structure of the domain itself (Balic et al., 2021). Functionally, alternative splicing of *ADAMTS17* affected secretion of the shorter isoform and altered the autocatalytic properties of ADAMTS17 and potentially the recognition of ADAMTS17 substrates. However, the *in vivo* consequences of alternative *ADAMTS17* splicing will need to be determined in future experiments. Besides the questions surrounding altered functions of the ADAMTS protease isoforms, how ADAMTS isoform generation through alternative splicing itself is regulated could warrant further investigation (Goncalves et al., 2017). This could be of special interest since alternative splicing events can be targeted with antisense oligonucleotides. For example exon-skipping therapy could potentially prevent the generation of detrimental *ADAMTS4* isoforms without affecting other functions of ADAMTS4 (Dzierlega and Yokota, 2020). Such an approach however, requires the exact knowledge about which ADAMTS domains are involved in the recognition of and binding to individual substrates.

### Proteolytic Processing of ADAMTS Proteases

ADAMTS proteases are initially synthesized as inactive zymogens and generally require a furin/proprotein convertase-mediated cleavage event that results in the removal of the propeptide, which then activates the protease. Most ADAMTS proteases contain one or more furin-consensus sites in their propeptide, with the one closest to the catalytic domain being cleaved during the activation step. If furin-processing at the other sites is required for zymogen maturation of ADAMTS proteases, as it was suggested for members of the related ADAM protease family, is unclear (Wong et al., 2015). For ADAMTS1 and ADAMTS5 it was shown that only one of the furin consensus sites was cleaved, while there is *in vitro* evidence for processing at the additional furin sites of ADAMTS9 and ADAMTS17 (Longpre and Leduc, 2004; Koo et al., 2007; Longpre et al., 2009; Hubmacher et al., 2017). The subcellular localization for furin-mediated activation differs for individual ADAMTS proteases. ADAMTS1 and ADAMTS4 are activated in the Golgi apparatus during secretion and the active enzyme was released into the cell culture medium (Longpre and Leduc, 2004; Wang et al., 2004). In contrast, ADAMTS5 was activated after secretion since mature ADAMTS5 was only detected in conditioned medium but not in cell lysate (Longpre et al., 2009). A third possible location for furin processing was described for ADAMTS9, where the zymogen was detected at the cell surface and mature ADAMTS9 was released into the medium after furin processing (Koo and Apte, 2010). ADAMTS7 was shown to be processed inside the cell, likely in the secretory pathway, and at the cell surface (Somerville et al., 2004b). The biological relevance of these different subcellular locations of zymogen processing remains unclear, but it could suggest that some ADAMTS proteases may cleave respective substrates already in the secretory pathway (ADAMTS1, ADAMTS4) while the activity of other ADAMTS proteases may be detrimental in this location and activation occurs outside of the cell restricting proteolytic activity to the cell surface or the ECM (ADAMTS5, ADAMTS9).

Exceptions to the generalized observation that propeptide removal is required to activate ADAMTS proteases are ADAMTS9, 10, 13, and 17. For ADAMTS9 it was shown that the pro-form, obtained by mutating all three furin sites or using a proprotein convertase inhibitor, was able to cleave its substrate versican even more efficiently then mature furin-processed ADAMTS9 and it was proposed that furin-processing of ADAMTS9 reduced its catalytic activity towards versican (Koo et al., 2007). Since the ADAMTS9 propeptide remained attached to the mature enzyme and a construct lacking the propeptide was not secreted at all, it was suggested that the ADAMTS9 propeptide was acting as a potential chaperone. In a very similar manner, ADAMTS17 retained its autocatalytic properties when the key furin processing site was mutated and ADAMTS17 secretion was abolished when the propeptide sequence was removed (Hubmacher et al., 2017). The sequence of ADAMTS13 includes only a very short propeptide, which does not seem to play a role as a chaperone during secretion or in modulating the catalytic activity of ADAMTS13 (Majerus et al., 2003). ADAMTS10 undergoes very inefficient furin processing due to the presence of a degenerated furin consensus sequence at the critical junction of the propeptide domain and the catalytic domain (Somerville et al., 2004a). Poorly furin-processed ADAMTS10 cleaved fibrillin-1 inefficiently *in vitro* (Kutz et al., 2011). However, upon restoration of the furin-consensus sequence by mutagenesis ADAMTS10 was activated, fibrillin-1 cleavage was enhanced and fibrillin-2 was now also cleaved (Wang et al., 2019a). Together with evidence that ADAMTS10 was size-shifted by α2-macroglobulin, which requires protease activity, ADAMTS10 could potentially work as a true protease if activated, potentially by non-furin proteases (Somerville et al., 2004a). However, if ADAMTS10 indeed functions as a true protease *in vivo* or through protease-independent mechanisms, such as modulation of fibrillin-1 assembly remains to be established (Kutz et al., 2011; Mularczyk et al., 2018; Wang et al., 2019a). With the advent of CRISPR/Cas9 gene editing, the introduction of point mutations in mice is greatly facilitated and it will be interesting to test the role of furin processing *in vivo* by introducing point mutations in furin processing sites or reestablishing a furin consensus site in *Adamts10*.

Autoproteolysis, the process of “self-cleavage” of ADAMTS proteases, adds an additional layer of complexity in the regulation of ADAMTS protease activity. Autocatalysis was described for several ADAMTS proteases including ADAMTS1, 4, 5, 7, and 17 with different consequences. Autocatalysis of ADAMTS1 results in two distinct ∼21 kDa peptides and can be prevented by the addition of heparin/heparan sulfates (Liu et al., 2006). ADAMTS4 can cleave itself in the Cys-rich and spacer domain through an intramolecular mechanism, resulting in distinct truncated ADAMTS4 proteases (Figure 3A) (Flannery et al., 2002). While the ability of truncated forms of ADAMTS4 to cleave bovine aggrecan were preserved, the affinities for sulfated glycosaminoglycans was reduced. This suggested the possibility that ECM-bound or cell surface-bound ADAMTS4 may be mobilized through autocatalysis, which now allows ADAMTS4 to reach its substrate aggrecan and contribute to its degradation in cartilage resulting in arthritis. Interestingly, similar proteolytic peptides were found for ADAMTS5 *in vitro* as well as in human cartilage and synovial tissue biopsies (Figure 3B) (Vankemmelbeke et al., 2001; Malfait et al., 2002; Zeng et al., 2006). It is currently unclear if these shortened ADAMTS5 isoforms were generated by autocatalytic processing or by ambient synovial proteases. Autoproteolysis of ADAMTS7 resulted in cleavage in the spacer domain, which may be involved in ADAMTS7 substrate recognition. It will be interesting to investigate, if the autocatalytic cleavage product of ADAMTS7 can still recognize its substrates (Colige et al., 2019). ADAMTS17 was autocatalytically cleaved in multiple locations, including in the catalytic domain itself and full-length mature ADAMTS17 was almost undetectable in conditioned medium (Hubmacher et al., 2017). In this case, autoproteolysis may be a mechanism to restrict ADAMTS17 protease activity to the cell surface or to generate bioactive peptides that are released from the cell surface. In addition to autocatalysis, ADAMTS proteases can be cleaved and/or activated by other proteases in the ECM. For example, ADAMTS4 was activated by MMP9, MMP13 and trypsin *in vitro* and ADAMTS1 was cleaved by MMP2, MMP8 and MMP15 (Rodriguez-Manzaneque et al., 2000; Gao et al., 2002; Tortorella et al., 2005). The relevance of these protease cascades *in vivo* remains to be established.

### Differential Cellular Localization of ADAMTS Proteases

ADAMTS-mediated substrate cleavage can only occur when ADAMTS proteases and their respective substrates are in close vicinity. Therefore, restricting ADAMTS protease activity to distinct cellular or subcellular compartments or signaling-induced relocation of ADAMTS proteases could shift their accessibility to different sets of substrates. As previously described, alternative splicing and autoproteolysis of ADAMTS4 could result in its release from the ECM (Gao et al., 2004). Additionally, alternative splicing and extensive autoproteolysis can spatially restrict ADAMTS17 protease activity to the cell surface, the adjacent pericellular matrix or an intracellular compartment that has yet to be identified (Hubmacher et al., 2017; Balic et al., 2021). The activation of ADAMTS5 outside of the cell may restrict its activity to the ECM and prevent substrate cleavage at the cell surface (Longpre et al., 2009). However, additional versican proteolysis by ADAMTS5 in the pericellular matrix was also reported (Hattori et al., 2011; Stupka et al., 2013).

ADAMTS protease activity can also be targeted to the ECM by binding of ADAMTS proteases to individual ECM proteins. This was demonstrated for example for ADAMTS1, 9, 10, and 17, which localized to fibronectin and fibrillin microfibrils in the ECM of cultured cells (Kuno and Matsushima, 1998; Kutz et al., 2011; Hubmacher et al., 2017; Wang et al., 2019b; Balic et al., 2021). In addition, ECM localization by binding to heparan-sulfate proteoglycans was demonstrated for ADAMTS1, 2, 4, and 5 (Kuno and Matsushima, 1998; Colige et al., 2005; Gendron et al., 2007; Fushimi et al., 2008). In these cases, addition of heparin to cells expressing the respective recombinant ADAMTS proteases resulted in their release into the medium. For ADAMTS2 it was shown that heparin increased the affinity of ADAMTS2 for TIMP3 (Wang et al., 2006). In the case of ADAMTS4 and ADAMTS5 addition of heparin inhibited the aggrecanases activity without affecting ADAMTS4 activity against other substrates (Vankemmelbeke et al., 2001; Fushimi et al., 2008). It will be interesting to see if binding of ADAMTS4 and ADAMTS5 to heparan-sulfate or other types of proteoglycans indeed inhibits aggrecanase activity *in vivo* and if this is a general property of the proteoglycanase subgroup to modulate substrate-specificity.

In an interesting experimental model ADAMTS9 was unintentionally trapped at the cell surface (Nandadasa et al., 2015). As a result, ADAMTS9 protease activity was restricted to the pericellular matrix while absent in the ECM. Several phenotypes were lacking when these mice were compared to the full ADAMTS9 knockout, suggesting that ADAMTS9 substrates in the pericellular matrix and the ECM are distinct and that ADAMTS9 is required in both compartments for normal development and tissue homeostasis. In addition, ADAMTS9 was identified inside the cell at the base of the primary cilium (Nandadasa et al., 2019). However, ADAMTS9 was not diverted from the secretory pathway, but was first secreted and then re-internalized. Since a short cilium phenotype could be restored by transfection with ADAMTS9, but not catalytically inactive ADAMTS9, a proteolytic function for ADAMTS9 in ciliogenesis was suggested.

The cell-surface receptor low-density lipoprotein receptor-related protein 1 (LRP1) was invoked as a mechanism for the re-uptake of secreted ADAMTS9 (Nandadasa et al., 2019). This is consistent with the LRP1-mediated uptake of ADAMTS4, ADAMTS5 and TIMP3, which has been described previously (Scilabra et al., 2013; Yamamoto et al., 2013; Yamamoto et al., 2014). In contrast to ADAMTS9, where LRP1-mediated uptake results in the shuttling of active ADAMTS9 to a new intracellular destination, it is thought that LRP1-mediated uptake of ADAMTS4 and ADAMTS5 is important to clear ADAMTS4 and ADAMTS5 from the cellular microenvironment and thus reduce, for example aggrecanase activity in cartilage. Interestingly, the domain requirements for binding to LRP1 differ between ADAMTS4 (cysteine-rich and spacer domain) and ADAMTS5 (spacer and TSR domain) and ADAMTS5 can compete with ADAMTS4 for binding to LRP1 (Yamamoto et al., 2014). In addition, it was demonstrated that LRP1 can be shed by ADAM17 and MMP14 in cartilage and the resulting soluble form of LRP1 prevented the uptake of ADAMTS5 without interfering with its catalytic activity. Since LRP1 shedding was increased in osteoarthritis, soluble LRP1 further augmented the aggrecanase activity in cartilage by preventing its cellular uptake (Yamamoto et al., 2017). As such, LRP1 and potentially other cell-surface receptors, are important regulators of ADAMTS recycling and the modulation of extracellular ADAMTS protease activity.

An unusual intracellular localization was described for ADAMTS1, which was found in the nucleus of a normal mammary cell line and two breast cancer cell lines (Silva et al., 2016). Since it was found together with aggrecan, the authors suggested that ADAMTS1 may play a proteolytic role in the nucleus. However, no nuclear substrates have been reported. A possible function for secreted ADAMTS proteases in intracellular compartments, such as the nucleus, raises many interesting questions, which have been partially answered for several MMPs that were identified in different intracellular compartments (Jobin et al., 2017).

Together, restricting ADAMTS protease activity to distinct subcellular and extracellular compartments represents an interesting way to regulate protease activity and substrate cleavage depending on the specific location. Conceptually, it is also possible that binding of ADAMTS proteases to distinct ECM proteins regulates the activity of these proteases by modulating binding affinities to individual substrates or by blocking access to substrate binding sites, adding yet an additional layer of complexity in spatially regulating ADAMTS protease activity.

## Inhibitors of ADAMTS Proteases

There has been a great interest in developing inhibitors to explore the feasibility of targeting ADAMTS protease isotypes to alter disease progression where aberrant protease activity results in tissue destruction (Yang et al., 2017; Santamaria, 2020). Limiting ADAMTS protease activity can in principle be achieved by decreasing ADAMTS protease expression, preventing ADAMTS protease activation, blocking their proteolytic activity or preventing the cleavage of specific ADAMTS substrates. Transcriptional targeting through modulation of signaling pathways or targeting of the proprotein convertase-mediated activation step are likely non-selective and will reduce the protease activity of several, if not all ADAMTS proteases with possible undesired side effects. Therefore, selectively targeting individual ADAMTS proteases with pharmacological or biological inhibitors, or preventing the cleavage of individual ADAMTS protease substrates will probably represent the most promising approaches to reduce ADAMTS protease activity in pathological settings. The most fruitful ventures in this field have been through the discovery and study of both endogenous and pharmacological inhibitors of specific ADAMTS proteases. Therefore, we will first describe the regulation of ADAMTS protease activity by endogenous inhibitors, focusing on tissue inhibitor of metalloproteinases (TIMP) 3 and α2-macroglobulin (α2M) and then summarize current strategies aimed at developing pharmacological inhibitors for specific ADAMTS proteases and substrate cleavage events. We will conclude with a summary of the clinical trials conducted for some of these inhibitors.

### Endogenous ADAMTS Protease Inhibitors

Homeostatic net protease activity in tissues can be controlled by carefully balancing the amount of proteases with the amount of endogenous protease inhibitors. An excess amount of proteases will lead to increased anabolic or catabolic ECM proteolysis while increasing the amount of protease inhibitors, such as TIMPs, will decrease or stop ECM proteolysis and protect tissues from excessive ECM turnover. There are four TIMP isotypes, which can inhibit virtually all metalloproteinases including MMPs, the pericellular ADAM proteases and ADAMTS proteases, albeit with different specificities and efficiencies for the individual protease families (Brew and Nagase, 2010; Arpino et al., 2015; Fan and Kassiri, 2020). For ADAMTS proteases, TIMP3 appears to be the most potent TIMP *in vivo*. In seminal biochemical studies it was shown that TIMP3 can inhibit ADAMTS4 and ADAMTS5 (Kashiwagi et al., 2001). A protective role for TIMP3 in articular cartilage homeostasis was identified in TIMP3 knockout mice and supports a role for TIMP3 as an important endogenous inhibitor for ADAMTS4 and ADAMTS5 (Sahebjam et al., 2007). More recently, transgenic overexpression of TIMP3 showed a protective effect toward articular cartilage degradation in a surgical osteoarthritis model (Nakamura et al., 2020). Interestingly, transgenic overexpression of a mutant TIMP3, which included an extra alanine-residue in its N-terminus that shifted the inhibitory profile for TIMP3 from “MMP plus ADAMTS” to a more selective “ADAMTS only” inhibition was even more efficient in preventing articular cartilage degradation (Lim et al., 2010; Nakamura et al., 2020). It will be fascinating to see how far the specificity of TIMP3 for ADAMTS4 and ADAMTS5 can be increased through systematic mutagenesis screens without losing the overall potency of TIMP3 as an ADAMTS protease inhibitor. More recently, TIMP4 was shown to inhibit ADAMTS7 even more efficiently then TIMP2 or TIMP3 (Colige et al., 2019). This raises the possibility that TIMP2 and TIMP4 are inhibitors for specific ADAMTS protease and the TIMP inhibition profile of ADAMTS proteases may need to be investigated on a protease-by-protease basis.

α2M is a large glycoprotein that is present in serum and tissues and that can inhibit almost all types of proteases in a “bait-and-trap” mechanism (Cuellar et al., 2016). The fact that α2M remains associated with proteases after cleavage, including the ADAMTS proteases, can be used to determine protease activity by gel-shift assays (Somerville et al., 2004a; Somerville et al., 2004b; Tortorella et al., 2004). ADAMTS1 was the first ADAMTS protease shown to bind, cleave and be inhibited by α2M (Kuno et al., 1999). Among ADAMTS proteases involved in arthritis, α2M was shown to inhibit both ADAMTS4 and ADAMTS5 in a dose-dependent manner (Tortorella et al., 2004). In addition, the cartilage oligomeric matrix protein (COMP)-processing activities of ADAMTS7 and ADAMTS12 were inhibited by α2M, suggesting that dysregulation of the ADAMTS7 and ADAMTS12 protease activity through α2M could be involved in arthritis (Luan et al., 2008). In the context of arthritis, α2M was found in similar concentrations as TIMP3 in the synovial fluid of joints and it rapidly bound to collagenase (Cawston et al., 1987). α2M was then identified as a key regulator of several cartilage degenerating factors and intra-articular injections of α2M could ameliorate osteoarthritis progression (Wang et al., 2014). In a similar approach as mentioned for TIMP3, the sequence of α2M was altered to increase its protective efficacy against cartilage degradation (Zhang et al., 2017). However, since α2M has many functions it is unclear to what extent the arthritis-protective effects can be related to the direct inhibition of ADAMTS proteases or the interaction of α2M with inflammatory cytokines, such as IL-1β or tumor necrosis factor TNFα (Wollenberg et al., 1991; Legres et al., 1994; Rehman et al., 2013).

In summary, it can be inferred that the presence of both TIMP3 and α2M are vital endogenous regulatory factors limiting ADAMTS protease activity within the joint and various other tissues. Any homeostatic imbalance between these two endogenous inhibitors and ADAMTS proteases can possibly lead to the onset or acceleration of degenerative diseases resulting in tissue destruction. Conversely, TIMP3 and α2M could potentially be used as a template to develop ADAMTS isotype specific peptide inhibitors.

### Pharmacological Inhibitors

The development of inhibitors for ADAMTS proteases is primarily focused on inhibiting ADAMTS4 and ADAMTS5 due to their prominent role in cartilage destruction in arthritis where both proteases degrade aggrecan, a major structural proteoglycan in the articular cartilage (Verma and Dalal, 2011). Conceptually, to inhibit ADAMTS4 and ADAMTS5, one could block their active site, prevent their binding to aggrecan or promote removal from the target tissue. All three approaches have been or are actively pursued in the quest to develop a specific inhibitor of aggrecanase activity to halt joint erosion in arthritis. However, a major complication in directly targeting ADAMTS4 and ADAMTS5 protease activity with small molecules is the high degree of sequence and structural conservation of the active site, not only within members of the ADAMTS protease family, but also between the ADAMTS, MMP, and ADAM protease families (Figure 1C) (Yiotakis and Dive, 2008; Kelwick et al., 2015). Therefore, unintended cross-inactivation of several metalloproteinases with possible short- and long-term side effects are likely when inhibitors are administered systemically. This may have been one of the reasons why early MMP inhibitors have failed as cancer therapeutics in clinical trials (Coussens et al., 2002; Winer et al., 2018). However, an ADAMTS5-targeting monoclonal antibody (mAb) was developed that bound at the interface of the catalytic and the disintegrin-like domain and appeared to reduce the structural flexibility of the active site, thus reducing ADAMTS5 activity (Figure 3B) (Larkin et al., 2015). The binding site of this ADAMTS5 mAb does not coincide with the aggrecan/versican substrate recognition site, which is located in the cysteine-rich and spacer domains (Santamaria et al., 2019). Very recently, a Zn-coordinating small molecule active site inhibitor was described for ADAMTS5 (GLPG 1972) that displayed strong selectivity against ADAMTS1 and other MMPs with the exception of ADAMTS4, where the selectivity was only ∼8 fold (Brebion et al., 2021). This selectivity is much lower compared to mAB-based exosite inhibitors against ADAMTS5, which did not bind to ADAMTS4 nor did they inhibit the protease or aggrecanase activity of ADAMTS4 (Santamaria et al., 2015). However, simultaneous inhibition of ADAMTS4 and ADAMTS5 may be desired in modifying arthritis progression. The ADAMTS5-inhibiting activity of GLPG1972 in articular cartilage has been studied extensively in both mouse and human cartilage tissue explants. In these studies, GLPG1972 inhibition of ADAMTS5 substantially reduced proteoglycan loss from femorotibial cartilage. Based on its performance in prior studies, GLPG1972 displays potent and selective inhibition of ADAMTS5, which may protect cartilage. However, a recently completed phase two clinical trial with GLPG1972 yielded disappointing results (see below).

As an alternative to targeting the active site the binding of ADAMTS proteases to its substrate could be blocked. The feasibility of such a strategy is further encouraged by the finding that epitopes in the ADAMTS ancillary domain, which is catalytically inactive and diverse in domain composition, contribute to substrate recognition through so-called “exosites” (Chiusaroli et al., 2013; Larkin et al., 2015; Santamaria et al., 2015). For example, it was shown that ADAMTS5 lacking the C-terminal ancillary domain was a poor aggrecanase when compared to full-length ADAMTS5 (Fushimi et al., 2008). In an extreme case, substrate preference for ADAMTS5 was switched from aggrecan to von Willebrand factor when its ancillary domain was swapped with the ancillary domain of ADAMTS13, the native ADAMTS protease that cleaves von Willebrand factor (Gao et al., 2012). However, the aggrecanase activity of ADAMTS5 could not be transferred to ADAMTS13 by fusing the ADAMTS5 ancillary domain with the ADAMTS13 catalytic domain. This may be explained by an unusual latent structure of the ADAMTS13 protease domain that requires allosteric activation by binding to von Willebrand factor, which differs from the ADAMTS5 protease domain (Petri et al., 2019). This suggests that not only the ancillary domain of ADAMTS5 but also the catalytic domain contributes specificity to substrate recognition and cleavage. Based on this rationale, a phage display screen identified mAbs against ADAMTS4 and ADAMTS5, which reacted with the catalytic/disintegrin domain outside of the active site, the spacer domain or the interface between the catalytic/disintegrin domain and the first TSR domain (Figure 3B) (Larkin et al., 2015; Santamaria et al., 2015). These mAbs reduced ADAMTS5 protease activity measured through aggrecan degradation. Additionally, when testing possible synergistic effects of combining both ADAMTS4- and ADAMTS5-inhibiting mAbs, ADAMTS5 appeared to be the dominant ADAMTS protease mediating cartilage degeneration in human cartilage explants or in primary human chondrocytes. As an alternative to mAbs, an ADAMTS5 exosite inhibitor based on a glycoconjugated arylsulfonamide was developed recently, which showed selectivity over ADAMTS4 and inhibited both, versican and aggrecan cleavage (Santamaria et al., 2021). It will be interesting to see how these inhibitors will perform *in vivo* since both, versicanase and aggrecanase activity, are blocked, or if exosite inhibitors can be developed that can discriminate between versican and aggrecan recognition.

A third strategy targeted the endogenous clearance pathway of ADAMTS4 and ADAMTS5, which involves LRP1. Both ADAMTS proteases are rapidly cleared in cartilage explants and articular chondrocytes by LRP1-mediated endocytosis (Yamamoto et al., 2013; Yamamoto et al., 2014). The interaction of ADAMTS4 and ADAMTS5 with LRP1 is mediated by the TSR1 domain and the spacer domain. Blockage of ADAMTS4 and ADAMTS5 clearance with an inhibitor resulted in increased aggrecan degradation. Interestingly, shedding of LRP1 was increased in osteoarthritic cartilage tissue, resulting in enhanced aggrecanase activity and inhibition of LRP1 shedding could potentially reduce degradation of cartilage ECM (Yamamoto et al., 2017). More recently, a role for TIMP3 in promoting LRP1-based protease clearance raises the possibility of identifying peptides that promote both, the binding and clearance of destructive ADAMTS proteases in cartilage to modify disease progression in arthritis (Carreca et al., 2020).

### Pharmaceutical Aggrecanase Inhibitor Projects and Clinical Trials

Several clinical trials have been conducted to test the safety and efficacy of arthritis-modifying candidate drugs that reduced aggrecanase activity, i.e., inhibited ADAMTS4 and ADAMTS5 in preclinical studies. However, none of these candidate drugs have advanced beyond phase II trials. The first aggrecanase inhibitor (AGG-523, Wyeth/Pfizer) was evaluated in phase I clinical trials (NCT00454298, NCT00454298) but results were not reported and further development of AGG-523 was discontinued, probably due to poor pharmacokinetics (Chockalingam et al., 2011; Malfait and Tortorella, 2019). Another small molecule inhibitor for ADAMTS5 (GLPG 1972, Galapagos) was tested in phase I/II trials (e.g. NCT03595618, NCT04136327) (Brebion et al., 2021). However, the phase II ROCCELLA trial (NCT03595618) with cartilage thickness as the primary endpoint did not improve outcome in patients with osteoarthritis (ThePharmaLetter, 2021). Several antibodies that are in commercial drug development pipelines were tested in phase I clinical studies. Safety and dose escalation studies were recently completed for an ADAMTS5 nanobody (M6495, Merck KGaA) (NCT03583346, NCT03224702) demonstrating safety, tolerability and a reduction in aggrecan proteolysis (Siebuhr et al., 2020). Further phase II clinical trials will be performed by Novartis (Merck, 2021). A humanized mAb (GSK2394002, GlaxoSmithKline), which was successful in modifying arthritis in preclinical studies, did not reach the clinical trial stage primarily due to unexpected and sustained cardiovascular side effects after a single dose injection of GSK2394002 in cynomolgus monkeys (Larkin et al., 2014; Larkin et al., 2015). An ADAMTS5 antibody against an epitope in the ancillary domain (CRB0017, Rottapharm) also ameliorated arthritis disease progression in knee joints of mice (Chiusaroli et al., 2013). However, the antibody is currently not listed as part of the Rottapharm drug pipeline and its clinical fate is unclear (RottapharmBiotech, 2021).

### Novel Technologies to Regulate ADAMTS Protease Activity

RBM-010 is an ADAMTS5-inhibiting aptamer developed by Ribomic, which awaits preclinical and clinical testing (Ribomic, 2021). Aptamers are single stranded RNA-based molecules that are selected from a randomized pool by their high binding affinity to a target protein and that have several advantages over other biomolecules as therapeutics (Zhang et al., 2019). An alternative method to reduce ADAMTS5 protease activity is the use of inhibitory RNA technologies to selectively degrade *ADAMTS5* mRNA. This could be achieved by delivering stabilized *ADAMTS5* antisense oligonucleotides directly into the knee joint. As a proof of principle, stabilized siRNA targeting *ADAMTS5* mRNA was injected into the nucleus pulposus after intervertebral disc puncture injury and subsequently delayed progression of intervertebral disc degeneration (Seki et al., 2009). More recently, a fibrin-hyaluronic acid hydrogel-based delivery system for *ADAMTS5* antisense oligonucleotides was described with the goal to develop a cartilage repair tissue that would inhibit ADAMTS5 activity within the co-delivered chondrocytes and the surrounding tissue (Garcia et al., 2019). It remains to be established if inhibitory RNA-based ADAMTS5 inactivation can modulate arthritis progression *in vivo*. CRISPR/Cas9-mediated gene deletion is an attractive approach to specifically delete target genes in select tissues. In combination with adeno-associated virus (AAV)-mediated gene delivery, one could envision that ADAMTS5 activity could be significantly reduced in knee joints affected by arthritis and halt disease progression (Evans et al., 2018; Fitzgerald, 2020). In a recent study, disease modification was described in a mouse model for osteoarthritis where CRISPR/Cas9-mediated simultaneous deletion of *NGF*, *IL-1β* and *MMP13* was achieved (Zhao et al., 2019). However, *ADAMTS5* was not targeted directly, but its gene expression was reduced.

## Conclusion and Outlook

In this review, we showcased the complexity of ADAMTS protease regulation, which ranges from transcriptional regulation to protease-mediated protease activation to balancing proteases and their endogenous inhibitors. However, several remaining questions need to be addressed in the future to fully understand the regulatory networks involving ADAMTS proteases and to fully harness the potential of inhibiting individual ADAMTS proteases to modify outcomes in degenerative diseases such as arthritis. For example, there is an ongoing quest to identify and validate the entire *in vivo* substrate spectrum for each individual ADAMTS protease and to determine the consequences of altered substrate cleavage (Schilling and Overall, 2007; Savickas and Auf dem Keller, 2017; Apte, 2020; Satz-Jacobowitz and Hubmacher, 2021). This is relevant to fully understand the biological function of individual proteases and to predict potential undesired side effects when designing isotype-specific inhibitors. An example is ADAMTS5, which can cleave aggrecan and versican but also the small leucine-rich proteoglycan decorin, biglycan and fibromodulin and possibly other proteins (Stanton et al., 2005; Gendron et al., 2007; Longpre et al., 2009). Some of these cleavage events may be beneficial and may need to be preserved in the presence of an ADAMTS5 inhibitor. In addition, inhibition of aggrecan cleavage to prevent tissue degradation may be desired in one tissue, such as the articular cartilage but simultaneously may have adverse effects in other tissues, such as tendon, where aggrecan accumulation resulted in decreased mechanical properties, or the aorta, where aggrecan and versican accumulated in thoracic aortic aneurysms potentially promoting aortic dissection and rupture (Velasco et al., 2011; Wang et al., 2012; Cikach et al., 2018). Therefore, an ideal small molecule or biologic inhibitor for ADAMTS5 as a disease modifying arthritis drug would be, one, highly specific for ADAMTS5; two, spare substrates other than aggrecan; and, three, deliverable to or become selectively activated in articular cartilage or the synovium. Since mAB 2B9 inhibited versicanase and aggrecanase activity of ADAMST5, the same exosites seem to be required for versican and aggrecan recognition and the design of such an “ideal” inhibitor may not be feasible.

Another question revolves around the regulatory networks in which ADAMTS proteases participate. These involve in one aspect transcriptional programs that induce distinct sets of genes, including ADAMTS proteases and that are regulated by specific signaling inputs, such as pro-inflammatory cytokines (Bondeson et al., 2008). By regulating critical nodes in the inflammatory cascade, ADAMTS protease could potentially be co-regulated together with other disease modifying genes. Additionally, it will be important to determine, if other ADAMTS sister proteases could compensate for an inhibited or downregulated ADAMTS protease as it was shown for ADAMTS9/ADAMTS20, ADAMTS7/ADAMTS12 or ADAMTS6/ADAMTS10 (McCulloch et al., 2009; Dubail et al., 2014; Mead et al., 2018; Mead et al., 2021).

Lastly, it will be interesting to explore novel technologies to regulate ADAMTS protease activity *in vivo*, for example by using antisense oligonucleotides, CRISPR/Cas-9 gene editing or by tissue-specific inhibitors. Such approaches may contribute to achieve the Holy Grail of ADAMTS isotype-specific and substrate-specific inhibitors that can then be deployed to modulate disease progression in degenerative conditions, such as arthritis.

## References

[B1] Alper M. Kockar F. (2014). IL-6 Upregulates a Disintegrin and Metalloproteinase with Thrombospondin Motifs 2 (ADAMTS-2) in Human Osteosarcoma Cells Mediated by JNK Pathway. Mol. Cell Biochem 393, 165–175. 10.1007/s11010-014-2056-9 24752352

[B2] Apte S. S. (2020). ADAMTS Proteins: Concepts, Challenges, and Prospects. Methods Mol. Biol. 2043, 1–12. 10.1007/978-1-4939-9698-8_1 31463898

[B3] Arpino V. Brock M. Gill S. E. (2015). The Role of TIMPs in Regulation of Extracellular Matrix Proteolysis. Matrix Biol. 44-46, 247–254. 10.1016/j.matbio.2015.03.005 25805621

[B4] Bai X.-H. Wang D.-W. Kong L. Zhang Y. Luan Y. Kobayashi T. (2009). ADAMTS-7, a Direct Target of PTHrP, Adversely Regulates Endochondral Bone Growth by Associating with and Inactivating GEP Growth Factor. Mol. Cell Biol 29, 4201–4219. 10.1128/mcb.00056-09 19487464 PMC2715794

[B5] Balic Z. Misra S. Willard B. Reinhardt D. P. Apte S. S. Hubmacher D. (2021). Alternative Splicing of the Metalloprotease ADAMTS17 Spacer Regulates Secretion and Modulates Autoproteolytic Activity. FASEB J. 35, e21310. 10.1096/fj.202001120rr 33484187 PMC8133003

[B6] Bekhouche M. Leduc C. Dupont L. Janssen L. Delolme F. Goff S. V. L. (2016). Determination of the Substrate Repertoire of ADAMTS2, 3, and 14 Significantly Broadens Their Functions and Identifies Extracellular Matrix Organization and TGF‐β Signaling as Primary Targets. FASEB J. 30, 1741–1756. 10.1096/fj.15-279869 26740262

[B7] Bevitt D. J. Li Z. Lindrop J. L. Barker M. D. Clarke M. P. Mckie N. (2005). Analysis of Full Length ADAMTS6 Transcript Reveals Alternative Splicing and a Role for the 5′ Untranslated Region in Translational Control. Gene 359, 99–110. 10.1016/j.gene.2005.06.011 16129570

[B8] Bevitt D. J. Mohamed J. Catterall J. B. Li Z. Arris C. E. Hiscott P. (2003). Expression of ADAMTS Metalloproteinases in the Retinal Pigment Epithelium Derived Cell Line ARPE-19: Transcriptional Regulation by TNFα. Biochim. Biophys. Acta (Bba) - Gene Struct. Expr. 1626, 83–91. 10.1016/s0167-4781(03)00047-2 12697333

[B9] Blelloch R. Anna-Arriola S. S. Gao D. Li Y. Hodgkin J. Kimble J. (1999). The Gon-1 Gene Is Required for Gonadal Morphogenesis in *Caenorhabditis elegans* . Dev. Biol. 216, 382–393. 10.1006/dbio.1999.9491 10588887

[B10] Blelloch R. Kimble J. (1999). Control of Organ Shape by a Secreted Metalloprotease in the Nematode *Caenorhabditis elegans* . Nature 399, 586–590. 10.1038/21196 10376599

[B11] Bondeson J. Wainwright S. Hughes C. Caterson B. (2008). The Regulation of the ADAMTS4 and ADAMTS5 Aggrecanases in Osteoarthritis: a Review. Clin. Exp. Rheumatol. 26, 139–145. 18328163

[B12] Brebion F. Gosmini R. Deprez P. Varin M. Peixoto C. Alvey L. (2021). Discovery of GLPG1972/S201086, a Potent, Selective, and Orally Bioavailable ADAMTS-5 Inhibitor for the Treatment of Osteoarthritis. J. Med. Chem. 64, 2937–2952. 10.1021/acs.jmedchem.0c02008 33719441

[B13] Brew K. Nagase H. (2010). The Tissue Inhibitors of Metalloproteinases (TIMPs): an Ancient Family with Structural and Functional Diversity. Biochim. Biophys. Acta (Bba) - Mol. Cell Res. 1803, 55–71. 10.1016/j.bbamcr.2010.01.003 PMC2853873 20080133

[B14] Brunet F. G. Fraser F. W. Binder M. J. Smith A. D. Kintakas C. Dancevic C. M. (2015). The Evolutionary Conservation of the A Disintegrin-like and Metalloproteinase Domain with Thrombospondin-1 Motif Metzincins across Vertebrate Species and Their Expression in Teleost Zebrafish. BMC Evol. Biol. 15, 22. 10.1186/s12862-015-0281-9 25879701 PMC4349717

[B15] Carreca A. P. Pravata V. M. Markham M. Bonelli S. Murphy G. Nagase H. (2020). TIMP-3 Facilitates Binding of Target Metalloproteinases to the Endocytic Receptor LRP-1 and Promotes Scavenging of MMP-1. Sci. Rep. 10, 12067. 10.1038/s41598-020-69008-9 32694578 PMC7374751

[B16] Cawston T. E. Mclaughlin P. Hazleman B. L. (1987). PAIRED SERUM AND SYNOVIAL FLUID VALUES OF α2-MACROGLOBULIN AND TIMP IN RHEUMATOID ARTHRITIS. Rheumatology 26, 354–358. 10.1093/rheumatology/26.5.354 2444303

[B17] Chiusaroli R. Visentini M. Galimberti C. Casseler C. Mennuni L. Covaceuszach S. (2013). Targeting of ADAMTS5's Ancillary Domain with the Recombinant mAb CRB0017 Ameliorates Disease Progression in a Spontaneous Murine Model of Osteoarthritis. Osteoarthritis and Cartilage 21, 1807–1810. 10.1016/j.joca.2013.08.015 23954517

[B18] Chockalingam P. S. Sun W. Rivera-Bermudez M. A. Zeng W. Dufield D. R. Larsson S. (2011). Elevated Aggrecanase Activity in a Rat Model of Joint Injury Is Attenuated by an Aggrecanase Specific Inhibitor. Osteoarthritis and Cartilage 19, 315–323. 10.1016/j.joca.2010.12.004 21163358

[B19] Choi G. C. G. Li J. Wang Y. Li L. Zhong L. Ma B. (2014). The Metalloprotease ADAMTS8 Displays Antitumor Properties through Antagonizing EGFR-MEK-ERK Signaling and Is Silenced in Carcinomas by CpG Methylation. Mol. Cancer Res. 12, 228–238. 10.1158/1541-7786.mcr-13-0195 24184540

[B20] Choi J. E. Kim D. S. Kim E. J. Chae M. H. Cha S. I. Kim C. H. (2008). Aberrant Methylation of ADAMTS1 in Non-small Cell Lung Cancer. Cancer Genet. Cytogenet. 187, 80–84. 10.1016/j.cancergencyto.2008.08.001 19027488

[B21] Cikach F. S. Koch C. D. Mead T. J. Galatioto J. Willard B. B. Emerton K. B. (2018). Massive Aggrecan and Versican Accumulation in Thoracic Aortic Aneurysm and Dissection. JCI Insight 3. 10.1172/jci.insight.97167 PMC5922288 29515038

[B22] Colige A. Monseur C. Crawley J. T. B. Santamaria S. De Groot R. (2019). Proteomic Discovery of Substrates of the Cardiovascular Protease ADAMTS7. J. Biol. Chem. 294, 8037–8045. 10.1074/jbc.ra119.007492 30926607 PMC6527163

[B23] Colige A. Ruggiero F. Vandenberghe I. Dubail J. Kesteloot F. Van Beeumen J. (2005). Domains and Maturation Processes that Regulate the Activity of ADAMTS-2, a Metalloproteinase Cleaving the Aminopropeptide of Fibrillar Procollagens Types I-III and V. J. Biol. Chem. 280, 34397–34408. 10.1074/jbc.m506458200 16046392

[B24] Colige A. Sieron A. L. Li S.-W. Schwarze U. Petty E. Wertelecki W. (1999). Human Ehlers-Danlos Syndrome Type VII C and Bovine Dermatosparaxis Are Caused by Mutations in the Procollagen I N-Proteinase Gene. Am. J. Hum. Genet. 65, 308–317. 10.1086/302504 10417273 PMC1377929

[B25] Coussens L. M. Fingleton B. Matrisian L. M. (2002). Matrix Metalloproteinase Inhibitors and Cancer--Trials and Tribulations. Science 295, 2387–2392. 10.1126/science.1067100 11923519

[B26] Cuéllar J. M. Cuéllar V. G. Scuderi G. J. (2016). α2-Macroglobulin. Phys. Med. Rehabil. Clin. North America 27, 909–918. 10.1016/j.pmr.2016.06.008 27788907

[B27] Dagoneau N. Benoist-Lasselin C. Huber C. Faivre L. Mégarbané A. Alswaid A. (2004). ADAMTS10 Mutations in Autosomal Recessive Weill-Marchesani Syndrome. Am. J. Hum. Genet. 75, 801–806. 10.1086/425231 15368195 PMC1182109

[B28] Doyle K. M. H. Russell D. L. Sriraman V. Richards J. S. (2004). Coordinate Transcription of the ADAMTS-1 Gene by Luteinizing Hormone and Progesterone Receptor. Mol. Endocrinol. 18, 2463–2478. 10.1210/me.2003-0380 15256533

[B29] Dubail J. Apte S. S. (2015). Insights on ADAMTS Proteases and ADAMTS-like Proteins from Mammalian Genetics. Matrix Biol. 44-46, 24–37. 10.1016/j.matbio.2015.03.001 25770910

[B30] Dubail J. Aramaki-Hattori N. Bader H. L. Nelson C. M. Katebi N. Matuska B. (2014). A newAdamts9conditional Mouse Allele Identifies its Non-redundant Role in Interdigital Web Regression. Genesis 52, 702–712. 10.1002/dvg.22784 24753090 PMC4107014

[B31] Dzierlega K. Yokota T. (2020). Optimization of Antisense-Mediated Exon Skipping for Duchenne Muscular Dystrophy. Gene Ther. 27, 407–416. 10.1038/s41434-020-0156-6 32483212

[B32] El Mabrouk M. Sylvester J. Zafarullah M. (2007). Signaling Pathways Implicated in Oncostatin M-Induced Aggrecanase-1 and Matrix Metalloproteinase-13 Expression in Human Articular Chondrocytes. Biochim. Biophys. Acta (Bba) - Mol. Cell Res. 1773, 309–320. 10.1016/j.bbamcr.2006.11.018 17208315

[B33] Enomoto H. Nelson C. M. Somerville R. P. T. Mielke K. Dixon L. J. Powell K. (2010). Cooperation of Two ADAMTS Metalloproteases in Closure of the Mouse Palate Identifies a Requirement for Versican Proteolysis in Regulating Palatal Mesenchyme Proliferation. Development 137, 4029–4038. 10.1242/dev.050591 21041365 PMC2976286

[B34] Evans C. H. Ghivizzani S. C. Robbins P. D. (2018). Gene Delivery to Joints by Intra-Articular Injection. Hum. Gene Ther. 29, 2–14. 10.1089/hum.2017.181 29160173 PMC5773261

[B35] Evans D. R. Green J. S. Fahiminiya S. Majewski J. Fernandez B. A. Deardorff M. A. (2020). A Novel Pathogenic Missense ADAMTS17 Variant that Impairs Secretion Causes Weill-Marchesani Syndrome with Variably Dysmorphic Hand Features. Sci. Rep. 10, 10827. 10.1038/s41598-020-66978-8 32616716 PMC7331723

[B36] Fan D. Kassiri Z. (2020). Biology of Tissue Inhibitor of Metalloproteinase 3 (TIMP3), and its Therapeutic Implications in Cardiovascular Pathology. Front. Physiol. 11, 661. 10.3389/fphys.2020.00661 32612540 PMC7308558

[B37] Fitzgerald J. (2020). Applications of CRISPR for Musculoskeletal Research. Bone Jt. Res. 9, 351–359. 10.1302/2046-3758.97.bjr-2019-0364.r2 PMC7357575 32676188

[B38] Flannery C. R. Zeng W. Corcoran C. Collins-Racie L. A. Chockalingam P. S. Hebert T. (2002). Autocatalytic Cleavage of ADAMTS-4 (Aggrecanase-1) Reveals Multiple Glycosaminoglycan-Binding Sites. J. Biol. Chem. 277, 42775–42780. 10.1074/jbc.m205309200 12202483

[B40] Fushimi K. Troeberg L. Nakamura H. Lim N. H. Nagase H. (2008). Functional Differences of the Catalytic and Non-catalytic Domains in Human ADAMTS-4 and ADAMTS-5 in Aggrecanolytic Activity. J. Biol. Chem. 283, 6706–6716. 10.1074/jbc.m708647200 18156631

[B41] Gao G. Plaas A. Thompson V. P. Jin S. Zuo F. Sandy J. D. (2004). ADAMTS4 (Aggrecanase-1) Activation on the Cell Surface Involves C-Terminal Cleavage by Glycosylphosphatidyl Inositol-Anchored Membrane Type 4-matrix Metalloproteinase and Binding of the Activated Proteinase to Chondroitin Sulfate and Heparan Sulfate on Syndecan-1. J. Biol. Chem. 279, 10042–10051. 10.1074/jbc.m312100200 14701864

[B42] Gao G. Westling J. Thompson V. P. Howell T. D. Gottschall P. E. Sandy J. D. (2002). Activation of the Proteolytic Activity of ADAMTS4 (Aggrecanase-1) by C-Terminal Truncation. J. Biol. Chem. 277, 11034–11041. 10.1074/jbc.m107443200 11796708

[B43] Gao S. De Geyter C. Kossowska K. Zhang H. (2007). FSH Stimulates the Expression of the ADAMTS-16 Protease in Mature Human Ovarian Follicles. Mol. Hum. Reprod. 13, 465–471. 10.1093/molehr/gam037 17519324

[B44] Gao W. Zhu J. Westfield L. A. Tuley E. A. Anderson P. J. Sadler J. E. (2012). Rearranging Exosites in Noncatalytic Domains Can Redirect the Substrate Specificity of ADAMTS Proteases. J. Biol. Chem. 287, 26944–26952. 10.1074/jbc.m112.380535 22707719 PMC3411030

[B45] Garcia J. P. Stein J. Cai Y. Riemers F. Wexselblatt E. Wengel J. (2019). Fibrin-hyaluronic Acid Hydrogel-Based Delivery of Antisense Oligonucleotides for ADAMTS5 Inhibition in Co-delivered and Resident Joint Cells in Osteoarthritis. J. Controlled Release 294, 247–258. 10.1016/j.jconrel.2018.12.030 30572032

[B46] Gendron C. Kashiwagi M. Lim N. H. Enghild J. J. Thøgersen I. B. Hughes C. (2007). Proteolytic Activities of Human ADAMTS-5. J. Biol. Chem. 282, 18294–18306. 10.1074/jbc.m701523200 17430884

[B47] Glasson S. S. Askew R. Sheppard B. Carito B. Blanchet T. Ma H.-L. (2005). Deletion of Active ADAMTS5 Prevents Cartilage Degradation in a Murine Model of Osteoarthritis. Nature 434, 644–648. 10.1038/nature03369 15800624

[B48] Goncalves V. Pereira J. F. S. Jordan P. (2017). Signaling Pathways Driving Aberrant Splicing in Cancer Cells. Genes (Basel) 9. 10.3390/genes9010009 PMC5793162 29286307

[B49] Hashimoto G. Shimoda M. Okada Y. (2004). ADAMTS4 (Aggrecanase-1) Interaction with the C-Terminal Domain of Fibronectin Inhibits Proteolysis of Aggrecan. J. Biol. Chem. 279, 32483–32491. 10.1074/jbc.m314216200 15161923

[B50] Hattori N. Carrino D. A. Lauer M. E. Vasanji A. Wylie J. D. Nelson C. M. (2011). Pericellular Versican Regulates the Fibroblast-Myofibroblast Transition. J. Biol. Chem. 286, 34298–34310. 10.1074/jbc.m111.254938 21828051 PMC3190794

[B51] Hofer T. P. J. Frankenberger M. Mages J. Lang R. Hoffmann R. Colige A. (2008). Tissue-specific Induction of ADAMTS2 in Monocytes and Macrophages by Glucocorticoids. J. Mol. Med. 86, 323–332. 10.1007/s00109-007-0284-0 18084737

[B52] Hu Z. Scott H. S. Qin G. Zheng G. Chu X. Xie L. (2015). Revealing Missing Human Protein Isoforms Based on Ab Initio Prediction, RNA-Seq and Proteomics. Sci. Rep. 5, 10940. 10.1038/srep10940 26156868 PMC4496727

[B53] Hubmacher D. Schneider M. Berardinelli S. J. Takeuchi H. Willard B. Reinhardt D. P. (2017). Unusual Life Cycle and Impact on Microfibril Assembly of ADAMTS17, a Secreted Metalloprotease Mutated in Genetic Eye Disease. Sci. Rep. 7, 41871. 10.1038/srep41871 28176809 PMC5296908

[B54] Huxley-Jones J. Apte S. S. Robertson D. L. Boot-Handford R. P. (2005). The Characterisation of Six ADAMTS Proteases in the Basal Chordate *Ciona intestinalis* Provides New Insights into the Vertebrate ADAMTS Family. Int. J. Biochem. Cell Biol. 37, 1838–1845. 10.1016/j.biocel.2005.03.009 15899586

[B55] Hynes R. O. (2012). The Evolution of Metazoan Extracellular Matrix. J. Cell Biol 196, 671–679. 10.1083/jcb.201109041 22431747 PMC3308698

[B56] Ilic M. Z. East C. J. Rogerson F. M. Fosang A. J. Handley C. J. (2007). Distinguishing Aggrecan Loss from Aggrecan Proteolysis in ADAMTS-4 and ADAMTS-5 Single and Double Deficient Mice. J. Biol. Chem. 282, 37420–37428. 10.1074/jbc.m703184200 17938173

[B57] Jacobi C. L. J. Rudigier L. J. Scholz H. Kirschner K. M. (2013). Transcriptional Regulation by the Wilms Tumor Protein, Wt1, Suggests a Role of the Metalloproteinase Adamts16 in Murine Genitourinary Development. J. Biol. Chem. 288, 18811–18824. 10.1074/jbc.m113.464644 23661704 PMC3696657

[B58] Jobin P. G. Butler G. S. Overall C. M. (2017). New Intracellular Activities of Matrix Metalloproteinases Shine in the Moonlight. Biochim. Biophys. Acta (Bba) - Mol. Cell Res. 1864, 2043–2055. 10.1016/j.bbamcr.2017.05.013 28526562

[B59] Jones G. C. Riley G. P. (2005). ADAMTS Proteinases: a Multi-Domain, Multi-Functional Family with Roles in Extracellular Matrix Turnover and Arthritis. Arthritis Res. Ther. 7, 160–169. 10.1186/ar1783 15987500 PMC1175049

[B60] Kapoor M. Martel-Pelletier J. Lajeunesse D. Pelletier J.-P. Fahmi H. (2011). Role of Proinflammatory Cytokines in the Pathophysiology of Osteoarthritis. Nat. Rev. Rheumatol. 7, 33–42. 10.1038/nrrheum.2010.196 21119608

[B61] Karoulias S. Z. Beyens A. Balic Z. Symoens S. Vandersteen A. Rideout A. L. (2020). A Novel ADAMTS17 Variant that Causes Weill-Marchesani Syndrome 4 Alters Fibrillin-1 and Collagen Type I Deposition in the Extracellular Matrix. Matrix Biol. 88, 1–18. 10.1016/j.matbio.2019.11.001 31726086

[B62] Kashiwagi M. Enghild J. J. Gendron C. Hughes C. Caterson B. Itoh Y. (2004). Altered Proteolytic Activities of ADAMTS-4 Expressed by C-Terminal Processing. J. Biol. Chem. 279, 10109–10119. 10.1074/jbc.m312123200 14662755

[B63] Kashiwagi M. Tortorella M. Nagase H. Brew K. (2001). TIMP-3 Is a Potent Inhibitor of Aggrecanase 1 (ADAM-TS4) and Aggrecanase 2 (ADAM-TS5). J. Biol. Chem. 276, 12501–12504. 10.1074/jbc.c000848200 11278243

[B64] Kelwick R. Desanlis I. Wheeler G. N. Edwards D. R. (2015). The ADAMTS (A Disintegrin and Metalloproteinase with Thrombospondin Motifs) Family. Genome Biol. 16, 113. 10.1186/s13059-015-0676-3 26025392 PMC4448532

[B65] Kim Y.-H. Lee H. C. Kim S.-Y. Yeom Y. I. Ryu K. J. Min B.-H. (2011). Epigenomic Analysis of Aberrantly Methylated Genes in Colorectal Cancer Identifies Genes Commonly Affected by Epigenetic Alterations. Ann. Surg. Oncol. 18, 2338–2347. 10.1245/s10434-011-1573-y 21298349 PMC3393129

[B66] Kleifeld O. Doucet A. Prudova A. auf dem Keller U. Gioia M. Kizhakkedathu J. N. (2011). Identifying and Quantifying Proteolytic Events and the Natural N Terminome by Terminal Amine Isotopic Labeling of Substrates. Nat. Protoc. 6, 1578–1611. 10.1038/nprot.2011.382 21959240

[B67] Koo B.-H. Apte S. S. (2010). Cell-surface Processing of the Metalloprotease Pro-ADAMTS9 Is Influenced by the Chaperone GRP94/gp96. J. Biol. Chem. 285, 197–205. 10.1074/jbc.m109.039677 19875450 PMC2804166

[B68] Koo B.-H. Longpré J.-M. Somerville R. P. T. Alexander J. P. Leduc R. Apte S. S. (2007). Regulation of ADAMTS9 Secretion and Enzymatic Activity by its Propeptide. J. Biol. Chem. 282, 16146–16154. 10.1074/jbc.m610161200 17403680

[B69] Kuno K. Matsushima K. (1998). ADAMTS-1 Protein Anchors at the Extracellular Matrix through the Thrombospondin Type I Motifs and its Spacing Region. J. Biol. Chem. 273, 13912–13917. 10.1074/jbc.273.22.13912 9593739

[B70] Kuno K. Terashima Y. Matsushima K. (1999). ADAMTS-1 Is an Active Metalloproteinase Associated with the Extracellular Matrix. J. Biol. Chem. 274, 18821–18826. 10.1074/jbc.274.26.18821 10373500

[B71] Kutz W. E. Wang L. W. Bader H. L. Majors A. K. Iwata K. Traboulsi E. I. (2011). ADAMTS10 Protein Interacts with Fibrillin-1 and Promotes its Deposition in Extracellular Matrix of Cultured Fibroblasts. J. Biol. Chem. 286, 17156–17167. 10.1074/jbc.m111.231571 21402694 PMC3089559

[B72] Larkin J. Lohr T. A. Elefante L. Shearin J. Matico R. Su J.-L. (2015). Translational Development of an ADAMTS-5 Antibody for Osteoarthritis Disease Modification. Osteoarthritis Cartilage 23, 1254–1266. 10.1016/j.joca.2015.02.778 25800415 PMC4516626

[B73] Larkin J. Lohr T. Elefante L. Shearin J. Matico R. Su J.-L. (2014). The Highs and Lows of Translational Drug Development: Antibody-Mediated Inhibition of ADAMTS-5 for Osteoarthritis Disease Modification. Osteoarthritis Cartilage 22, S483–S484. 10.1016/j.joca.2014.02.918 PMC4516626 25800415

[B74] Le Goff C. Somerville R. P. T. Kesteloot F. Powell K. Birk D. E. Colige A. C. (2006). Regulation of Procollagen Amino-Propeptide Processing during Mouse Embryogenesis by Specialization of Homologous ADAMTS Proteases: Insights on Collagen Biosynthesis and Dermatosparaxis. Development 133, 1587–1596. 10.1242/dev.02308 16556917

[B75] Leduc C. Dupont L. Joannes L. Monseur C. Baiwir D. Mazzucchelli G. (2021). *In Vivo* N-Terminomics Highlights Novel Functions of ADAMTS2 and ADAMTS14 in Skin Collagen Matrix Building. Front. Mol. Biosci. 8, 643178. 10.3389/fmolb.2021.643178 33816558 PMC8017238

[B76] Legrès L. G. Pochon F. Barray M. Heinrich P. C. Delain E. (1994). Human ?2-Macroglobulin as a Cytokine-Binding Plasma Protein. Ann. NY Acad. Sci. 737, 439–443. 10.1111/j.1749-6632.1994.tb44334.x 7524417

[B77] Li Y. (2021). Modern Epigenetics Methods in Biological Research. Methods 187, 104–113. 10.1016/j.ymeth.2020.06.022 32645449 PMC7785612

[B78] Lim N. H. Kashiwagi M. Visse R. Jones J. Enghild J. J. Brew K. (2010). Reactive-site Mutants of N-TIMP-3 that Selectively Inhibit ADAMTS-4 and ADAMTS-5: Biological and Structural Implications. Biochem. J. 431, 113–122. 10.1042/bj20100725 20645923 PMC3003256

[B79] Liu Y.-J. Xu Y. Yu Q. (2006). Full-length ADAMTS-1 and the ADAMTS-1 Fragments Display Pro- and Antimetastatic Activity, Respectively. Oncogene 25, 2452–2467. 10.1038/sj.onc.1209287 16314835 PMC2759703

[B80] Longpré J.-M. Leduc R. (2004). Identification of Prodomain Determinants Involved in ADAMTS-1 Biosynthesis. J. Biol. Chem. 279, 33237–33245. 10.1074/jbc.m313151200 15184385

[B81] Longpré J.-M. Mcculloch D. R. Koo B.-H. Alexander J. P. Apte S. S. Leduc R. (2009). Characterization of proADAMTS5 Processing by Proprotein Convertases. Int. J. Biochem. Cell Biol. 41, 1116–1126. 10.1016/j.biocel.2008.10.008 18992360

[B82] Luan Y. Kong L. Howell D. R. Ilalov K. Fajardo M. Bai X.-H. (2008). Inhibition of ADAMTS-7 and ADAMTS-12 Degradation of Cartilage Oligomeric Matrix Protein by Alpha-2-Macroglobulin. Osteoarthritis and Cartilage 16, 1413–1420. 10.1016/j.joca.2008.03.017 18485748 PMC2574789

[B83] Madeira F. Park Y. m. Lee J. Buso N. Gur T. Madhusoodanan N. (2019). The EMBL-EBI Search and Sequence Analysis Tools APIs in 2019. Nucleic Acids Res. 47, W636–W641. 10.1093/nar/gkz268 30976793 PMC6602479

[B84] Majerus E. M. Zheng X. Tuley E. A. Sadler J. E. (2003). Cleavage of the ADAMTS13 Propeptide Is Not Required for Protease Activity. J. Biol. Chem. 278, 46643–46648. 10.1074/jbc.m309872200 12975358 PMC11060746

[B85] Malfait A.-M. Liu R.-Q. Ijiri K. Komiya S. Tortorella M. D. (2002). Inhibition of ADAM-TS4 and ADAM-TS5 Prevents Aggrecan Degradation in Osteoarthritic Cartilage. J. Biol. Chem. 277, 22201–22208. 10.1074/jbc.m200431200 11956193

[B86] Malfait A. M. Tortorella M. D. (2019). The “elusive DMOAD”: Aggrecanase Inhibition from Laboratory to Clinic. Clin. Exp. Rheumatol. 37 (Suppl. 120), 130–134. 31621572

[B87] Mani S. Ghosh J. Lan Y. Senapati S. Ord T. Sapienza C. (2019). Epigenetic Changes in Preterm Birth Placenta Suggest a Role for ADAMTS Genes in Spontaneous Preterm Birth. Hum. Mol. Genet. 28, 84–95. 10.1093/hmg/ddy325 30239759 PMC6335625

[B88] McCulloch D. R. Nelson C. M. Dixon L. J. Silver D. L. Wylie J. D. Lindner V. (2009). ADAMTS Metalloproteases Generate Active Versican Fragments that Regulate Interdigital Web Regression. Dev. Cell 17, 687–698. 10.1016/j.devcel.2009.09.008 19922873 PMC2780442

[B89] Mead T. J. Apte S. S. (2018). ADAMTS Proteins in Human Disorders. Matrix Biol. 71-72, 225–239. 10.1016/j.matbio.2018.06.002 29885460 PMC6146047

[B90] Mead T. J. Mcculloch D. R. Ho J. C. Du Y. Adams S. M. Birk D. E. (2018). The Metalloproteinase-Proteoglycans ADAMTS7 and ADAMTS12 Provide an Innate, Tendon-specific Protective Mechanism against Heterotopic Ossification. JCI Insight 3. 10.1172/jci.insight.92941 PMC5928868 29618652

[B91] Mead T. J. Martin D. R. Wang L. W. Cain S. A. Gulec C. Cahill E. (2021). Proteolysis of Fibrillin-2 Microfibrils Is Essential for normal Skeletal Development. bioRxiv. 10.1101/2021.02.03.429587 PMC9064305 35503090

[B92] Merck (2021). Available at: www.emdgroup.com/en/news/out-licensing-anti-adamts5-06-10-2020.html. (Accessed 01 04, 2021).

[B93] Mimata Y. Kamataki A. Oikawa S. Murakami K. Uzuki M. Shimamura T. (2012). Interleukin-6 Upregulates Expression of ADAMTS-4 in Fibroblast-like Synoviocytes from Patients with Rheumatoid Arthritis. Int. J. Rheum. Dis. 15, 36–44. 10.1111/j.1756-185x.2011.01656.x 22324945

[B94] Moncada-Pazos A. Obaya A. J. Fraga M. F. Viloria C. G. Capellá G. Gausachs M. (2009). The ADAMTS12 Metalloprotease Gene Is Epigenetically Silenced in Tumor Cells and Transcriptionally Activated in the Stroma during Progression of colon Cancer. J. Cell Sci 122, 2906–2913. 10.1242/jcs.050468 19638407

[B95] Morales J. Al-Sharif L. Khalil D. S. Shinwari J. M. A. Bavi P. Al-Mahrouqi R. A. (2009). Homozygous Mutations in ADAMTS10 and ADAMTS17 Cause Lenticular Myopia, Ectopia Lentis, Glaucoma, Spherophakia, and Short Stature. Am. J. Hum. Genet. 85, 558–568. 10.1016/j.ajhg.2009.09.011 19836009 PMC2775842

[B96] Mularczyk E. J. Singh M. Godwin A. R. F. Galli F. Humphreys N. Adamson A. D. (2018). ADAMTS10-mediated Tissue Disruption in Weill-Marchesani Syndrome. Hum. Mol. Genet. 27, 3675–3687. 10.1093/hmg/ddy276 30060141 PMC6196651

[B97] Nakamura H. Vo P. Kanakis I. Liu K. Bou-Gharios G. (2020). Aggrecanase-selective Tissue Inhibitor of Metalloproteinase-3 (TIMP3) Protects Articular Cartilage in a Surgical Mouse Model of Osteoarthritis. Sci. Rep. 10, 9288. 10.1038/s41598-020-66233-0 32518385 PMC7283274

[B98] Nandadasa S. Kraft C. M. Wang L. W. O'donnell A. Patel R. Gee H. Y. (2019). Secreted Metalloproteases ADAMTS9 and ADAMTS20 Have a Non-canonical Role in Ciliary Vesicle Growth during Ciliogenesis. Nat. Commun. 10, 953. 10.1038/s41467-019-08520-7 30814516 PMC6393521

[B99] Nandadasa S. Nelson C. M. Apte S. S. (2015). ADAMTS9-Mediated Extracellular Matrix Dynamics Regulates Umbilical Cord Vascular Smooth Muscle Differentiation and Rotation. Cell Rep. 11, 1519–1528. 10.1016/j.celrep.2015.05.005 26027930 PMC4472575

[B100] Nicholson A. C. Malik S. B. Logsdon J. M. Jr. Van Meir E. G. (2005). Functional Evolution of ADAMTS Genes: Evidence from Analyses of Phylogeny and Gene Organization. BMC Evol. Biol. 5, 11. 10.1186/1471-2148-5-11 15693998 PMC549037

[B101] Petri A. Kim H. J. Xu Y. De Groot R. Li C. Vandenbulcke A. (2019). Crystal Structure and Substrate-Induced Activation of ADAMTS13. Nat. Commun. 10, 3781. 10.1038/s41467-019-11474-5 31439947 PMC6706451

[B102] Puente X. S. Sánchez L. M. Overall C. M. López-Otín C. (2003). Human and Mouse Proteases: a Comparative Genomic Approach. Nat. Rev. Genet. 4, 544–558. 10.1038/nrg1111 12838346

[B103] Rabadan R. Mohamedi Y. Rubin U. Chu T. Alghalith A. N. Elliott O. (2020). Identification of Relevant Genetic Alterations in Cancer Using Topological Data Analysis. Nat. Commun. 11, 3808. 10.1038/s41467-020-17659-7 32732999 PMC7393176

[B104] Redondo-García S. Peris-Torres C. Caracuel-Peramos R. Rodríguez-Manzaneque J. C. (2021). ADAMTS Proteases and the Tumor Immune Microenvironment: Lessons from Substrates and Pathologies. Matrix Biol. Plus 9, 100054. 10.1016/j.mbplus.2020.100054 33718860 PMC7930849

[B105] Rehman A. A. Ahsan H. Khan F. H. (2013). alpha-2-Macroglobulin: a Physiological Guardian. J. Cell. Physiol. 228, 1665–1675. 10.1002/jcp.24266 23086799

[B106] Ribomic (2021). Available at: www.ribomic.com/eng/pipeline.php. (Accessed 01 04, 2021).

[B107] Rodriguez-Manzaneque J. C. Milchanowski A. B. Dufour E. K. Leduc R. Iruela-Arispe M. L. (2000). Characterization of METH-1/ADAMTS1 Processing Reveals Two Distinct Active Forms. J. Biol. Chem. 275, 33471–33479. 10.1074/jbc.M002599200 10944521

[B108] RottapharmBiotech (2021). Available at: www.rottapharmbiotech.com/pipeline/#top. (Accessed 01 04, 2021).

[B109] Sadler J. E. (2008). Von Willebrand Factor, ADAMTS13, and Thrombotic Thrombocytopenic Purpura. Blood 112, 11–18. 10.1182/blood-2008-02-078170 18574040 PMC2435681

[B110] Sahebjam S. Khokha R. Mort J. S. (2007). Increased Collagen and Aggrecan Degradation with Age in the Joints of Timp3−/− Mice. Arthritis Rheum. 56, 905–909. 10.1002/art.22427 17328064

[B111] Santamaria S. (2020). ADAMTS‐5: A Difficult Teenager Turning 20. Int. J. Exp. Path. 101, 4–20. 10.1111/iep.12344 32219922 PMC7306899

[B112] Santamaria S. Cuffaro D. Nuti E. Ciccone L. Tuccinardi T. Liva F. (2021). Exosite Inhibition of ADAMTS-5 by a Glycoconjugated Arylsulfonamide. Sci. Rep. 11, 949. 10.1038/s41598-020-80294-1 33441904 PMC7806935

[B113] Santamaria S. Yamamoto K. Botkjaer K. Tape C. Dyson M. R. Mccafferty J. (2015). Antibody-based Exosite Inhibitors of ADAMTS-5 (Aggrecanase-2). Biochem. J. 471, 391–401. 10.1042/bj20150758 26303525 PMC4613496

[B114] Santamaria S. Yamamoto K. Teraz-Orosz A. Koch C. Apte S. S. De Groot R. (2019). Exosites in Hypervariable Loops of ADAMTS Spacer Domains Control Substrate Recognition and Proteolysis. Sci. Rep. 9, 10914. 10.1038/s41598-019-47494-w 31358852 PMC6662762

[B115] Satz-Jacobowitz B. Hubmacher D. (2021). The Quest for Substrates and Binding Partners: A Critical Barrier for Understanding the Role of ADAMTS Proteases in Musculoskeletal Development and Disease. Dev. Dyn. 250, 8–26. 10.1002/dvdy.248 32875613 PMC7902295

[B116] Savickas S. Auf dem Keller U. (2017). Targeted Degradomics in Protein Terminomics and Protease Substrate Discovery. Biol. Chem. 399, 47–54. 10.1515/hsz-2017-0187 28850541

[B117] Schilling O. Overall C. M. (2007). Proteomic Discovery of Protease Substrates. Curr. Opin. Chem. Biol. 11, 36–45. 10.1016/j.cbpa.2006.11.037 17194619

[B118] Schnellmann R. Sack R. Hess D. Annis D. S. Mosher D. F. Apte S. S. (2018). A Selective Extracellular Matrix Proteomics Approach Identifies Fibronectin Proteolysis by A Disintegrin-like and Metalloprotease Domain with Thrombospondin Type 1 Motifs (ADAMTS16) and its Impact on Spheroid Morphogenesis. Mol. Cell Proteomics 17, 1410–1425. 10.1074/mcp.ra118.000676 29669734 PMC6030725

[B119] Scilabra S. D. Troeberg L. Yamamoto K. Emonard H. Thøgersen I. Enghild J. J. (2013). Differential Regulation of Extracellular Tissue Inhibitor of Metalloproteinases-3 Levels by Cell Membrane-Bound and Shed Low Density Lipoprotein Receptor-Related Protein 1. J. Biol. Chem. 288, 332–342. 10.1074/jbc.m112.393322 23166318 PMC3537031

[B120] Seki S. Asanuma-Abe Y. Masuda K. Kawaguchi Y. Asanuma K. Muehleman C. (2009). Effect of Small Interference RNA (siRNA) for ADAMTS5 on Intervertebral Disc Degeneration in the Rabbit Anular Needle-Puncture Model. Arthritis Res. Ther. 11, R166. 10.1186/ar2851 19889209 PMC3003501

[B121] Shindo T. Kurihara H. Kuno K. Yokoyama H. Wada T. Kurihara Y. (2000). ADAMTS-1: a Metalloproteinase-Disintegrin Essential for normal Growth, Fertility, and Organ Morphology and Function. J. Clin. Invest. 105, 1345–1352. 10.1172/jci8635 10811842 PMC315464

[B122] Siebuhr A. S. Werkmann D. Bay-Jensen A. C. Thudium C. S. Karsdal M. A. Serruys B. (2020). The Anti-ADAMTS-5 Nanobody((R)) M6495 Protects Cartilage Degradation *Ex Vivo* . Int. J. Mol. Sci. 21, 5992. 10.3390/ijms21175992 PMC7503673 32825512

[B123] Silva S. V. Lima M. A. Cella N. Jaeger R. G. Freitas V. M. (2016). ADAMTS-1 Is Found in the Nuclei of Normal and Tumoral Breast Cells. PLoS One 11, e0165061. 10.1371/journal.pone.0165061 27764205 PMC5072708

[B124] Somerville R. P. T. Jungers K. A. Apte S. S. (2004a). Discovery and Characterization of a Novel, Widely Expressed Metalloprotease, ADAMTS10, and its Proteolytic Activation. J. Biol. Chem. 279, 51208–51217. 10.1074/jbc.m409036200 15355968

[B125] Somerville R. P. T. Longpré J.-M. Apel E. D. Lewis R. M. Wang L. W. Sanes J. R. (2004b). ADAMTS7B, the Full-Length Product of the ADAMTS7 Gene, Is a Chondroitin Sulfate Proteoglycan Containing a Mucin Domain. J. Biol. Chem. 279, 35159–35175. 10.1074/jbc.m402380200 15192113

[B126] Song R.-H. D. Tortorella M. Malfait A.-M. Alston J. T. Yang Z. Arner E. C. (2007). Aggrecan Degradation in Human Articular Cartilage Explants Is Mediated by Both ADAMTS-4 and ADAMTS-5. Arthritis Rheum. 56, 575–585. 10.1002/art.22334 17265492

[B127] Stankunas K. Hang C. T. Tsun Z.-Y. Chen H. Lee N. V. Wu J. I. (2008). Endocardial Brg1 Represses ADAMTS1 to Maintain the Microenvironment for Myocardial Morphogenesis. Dev. Cell 14, 298–311. 10.1016/j.devcel.2007.11.018 18267097 PMC2274005

[B128] Stanton H. Rogerson F. M. East C. J. Golub S. B. Lawlor K. E. Meeker C. T. (2005). ADAMTS5 Is the Major Aggrecanase in Mouse Cartilage *In Vivo* and *In Vitro* . Nature 434, 648–652. 10.1038/nature03417 15800625

[B129] Stupka N. Kintakas C. White J. D. Fraser F. W. Hanciu M. Aramaki-Hattori N. (2013). Versican Processing by a Disintegrin-like and Metalloproteinase Domain with Thrombospondin-1 Repeats Proteinases-5 and -15 Facilitates Myoblast Fusion. J. Biol. Chem. 288, 1907–1917. 10.1074/jbc.m112.429647 23233679 PMC3548499

[B130] Surridge A. K. Rodgers U. R. Swingler T. E. Davidson R. K. Kevorkian L. Norton R. (2009). Characterization and Regulation of ADAMTS-16. Matrix Biol. 28, 416–424. 10.1016/j.matbio.2009.07.001 19635554 PMC2789966

[B131] Takeda S. (2016). ADAM and ADAMTS Family Proteins and Snake Venom Metalloproteinases: A Structural Overview. Toxins (Basel) 8, 155. 10.3390/toxins8050155 PMC4885070 27196928

[B132] ThePharmaLetter (2021). Available at: https://www.thepharmaletter.com/article/galapagos-and-servier-s-glpg1972-s201086-fails-in-knee-osteoarthritis-patients. (Accessed 01 04, 2021).

[B133] Tian Y. Yuan W. Fujita N. Wang J. Wang H. Shapiro I. M. (2013). Inflammatory Cytokines Associated with Degenerative Disc Disease Control Aggrecanase-1 (ADAMTS-4) Expression in Nucleus Pulposus Cells through MAPK and NF-κB. Am. J. Pathol. 182, 2310–2321. 10.1016/j.ajpath.2013.02.037 23602832 PMC3668031

[B134] Tortorella M. D. Arner E. C. Hills R. Easton A. Korte-Sarfaty J. Fok K. (2004). α2-Macroglobulin Is a Novel Substrate for ADAMTS-4 and ADAMTS-5 and Represents an Endogenous Inhibitor of These Enzymes. J. Biol. Chem. 279, 17554–17561. 10.1074/jbc.m313041200 14715656

[B135] Tortorella M. D. Arner E. C. Hills R. Gormley J. Fok K. Pegg L. (2005). ADAMTS-4 (Aggrecanase-1): N-Terminal Activation Mechanisms. Arch. Biochem. Biophys. 444, 34–44. 10.1016/j.abb.2005.09.018 16289022

[B136] Uchida K. Takano S. Matsumoto T. Nagura N. Inoue G. Itakura M. (2017). Transforming Growth Factor Activating Kinase 1 Regulates Extracellular Matrix Degrading Enzymes and Pain-Related Molecule Expression Following Tumor Necrosis Factor-Alpha Stimulation of Synovial Cells: an *In Vitro* Study. BMC Musculoskelet. Disord. 18, 283. 10.1186/s12891-017-1648-4 28668088 PMC5493881

[B137] Vankemmelbeke M. N. Holen I. Wilson A. G. Ilic M. Z. Handley C. J. Kelner G. S. (2001). Expression and Activity of ADAMTS-5 in Synovium. Eur. J. Biochem. 268, 1259–1268. 10.1046/j.1432-1327.2001.01990.x 11231277

[B138] Velasco J. Li J. Dipietro L. Stepp M. A. Sandy J. D. Plaas A. (2011). Adamts5 Deletion Blocks Murine Dermal Repair through CD44-Mediated Aggrecan Accumulation and Modulation of Transforming Growth Factor β1 (TGFβ1) Signaling. J. Biol. Chem. 286, 26016–26027. 10.1074/jbc.m110.208694 21566131 PMC3138253

[B139] Verma P. Dalal K. (2011). ADAMTS-4 and ADAMTS-5: Key Enzymes in Osteoarthritis. J. Cell. Biochem. 112, 3507–3514. 10.1002/jcb.23298 21815191

[B140] Vistnes M. Aronsen J. M. Lunde I. G. Sjaastad I. Carlson C. R. Christensen G. (2014). Pentosan Polysulfate Decreases Myocardial Expression of the Extracellular Matrix Enzyme ADAMTS4 and Improves Cardiac Function *In Vivo* in Rats Subjected to Pressure Overload by Aortic Banding. Plos One 9, e89621. 10.1371/journal.pone.0089621 24595230 PMC3940660

[B141] Wagstaff L. Kelwick R. Decock J. Edwards D. R. (2011). The Roles of ADAMTS Metalloproteinases in Tumorigenesis and Metastasis. Front. Biosci. 16, 1861–1872. 10.2741/3827 21196270

[B142] Wainwright S. D. Bondeson J. Hughes C. E. (2006). An Alternative Spliced Transcript of ADAMTS4 Is Present in Human Synovium from OA Patients. Matrix Biol. 25, 317–320. 10.1016/j.matbio.2006.03.006 16723216

[B143] Wang L. W. Kutz W. E. Mead T. J. Beene L. C. Singh S. Jenkins M. W. (2019a). Adamts10 Inactivation in Mice Leads to Persistence of Ocular Microfibrils Subsequent to Reduced Fibrillin-2 Cleavage. Matrix Biol. 77, 117–128. 10.1016/j.matbio.2018.09.004 30201140 PMC8209899

[B144] Wang L. W. Nandadasa S. Annis D. S. Dubail J. Mosher D. F. Willard B. B. (2019b). A Disintegrin-like and Metalloproteinase Domain with Thrombospondin Type 1 Motif 9 (ADAMTS9) Regulates Fibronectin Fibrillogenesis and Turnover. J. Biol. Chem. 294, 9924–9936. 10.1074/jbc.ra118.006479 31085586 PMC6597835

[B145] Wang P. Tortorella M. England K. Malfait A.-M. Thomas G. Arner E. C. (2004). Proprotein Convertase Furin Interacts with and Cleaves Pro-ADAMTS4 (Aggrecanase-1) in the Trans-Golgi Network. J. Biol. Chem. 279, 15434–15440. 10.1074/jbc.m312797200 14744861

[B146] Wang S. Wei X. Zhou J. Zhang J. Li K. Chen Q. (2014). Identification of α2-Macroglobulin as a Master Inhibitor of Cartilage-Degrading Factors that Attenuates the Progression of Posttraumatic Osteoarthritis. Arthritis Rheumatol. 66, 1843–1853. 10.1002/art.38576 24578232 PMC4187342

[B147] Wang V. M. Bell R. M. Thakore R. Eyre D. R. Galante J. O. Li J. (2012). Murine Tendon Function Is Adversely Affected by Aggrecan Accumulation Due to the Knockout of ADAMTS5. J. Orthop. Res. 30, 620–626. 10.1002/jor.21558 21928430

[B148] Wang W.-M. Ge G. Lim N. H. Nagase H. Greenspan D. S. (2006). TIMP-3 Inhibits the Procollagen N-Proteinase ADAMTS-2. Biochem. J. 398, 515–519. 10.1042/bj20060630 16771712 PMC1559475

[B149] Winer A. Adams S. Mignatti P. (2018). Matrix Metalloproteinase Inhibitors in Cancer Therapy: Turning Past Failures into Future Successes. Mol. Cancer Ther. 17, 1147–1155. 10.1158/1535-7163.mct-17-0646 29735645 PMC5984693

[B150] Wollenberg G. K. Lamarre J. Rosendal S. Gonias S. L. Hayes M. A. (1991). Binding of Tumor Necrosis Factor Alpha to Activated Forms of Human Plasma Alpha 2 Macroglobulin. Am. J. Pathol. 138, 265–272. 1704186 PMC1886187

[B151] Wong E. Maretzky T. Peleg Y. Blobel C. P. Sagi I. (2015). The Functional Maturation of A Disintegrin and Metalloproteinase (ADAM) 9, 10, and 17 Requires Processing at a Newly Identified Proprotein Convertase (PC) Cleavage Site. J. Biol. Chem. 290, 12135–12146. 10.1074/jbc.m114.624072 25795784 PMC4424348

[B152] Wünnemann F. Ta-Shma A. Ta-Shma A. Preuss C. Leclerc S. van Vliet P. P. (2020). Loss of ADAMTS19 Causes Progressive Non-syndromic Heart Valve Disease. Nat. Genet. 52, 40–47. 10.1038/s41588-019-0536-2 31844321 PMC7197892

[B153] Xu Y. R. Lei C. Q. (2020). TAK1-TABs Complex: A Central Signalosome in Inflammatory Responses. Front. Immunol. 11, 608976. 10.3389/fimmu.2020.608976 33469458 PMC7813674

[B154] Yamamoto K. Owen K. Parker A. E. Scilabra S. D. Dudhia J. Strickland D. K. (2014). Low Density Lipoprotein Receptor-Related Protein 1 (LRP1)-Mediated Endocytic Clearance of a Disintegrin and Metalloproteinase with Thrombospondin Motifs-4 (ADAMTS-4). J. Biol. Chem. 289, 6462–6474. 10.1074/jbc.m113.545376 24474687 PMC3945312

[B155] Yamamoto K. Santamaria S. Botkjaer K. A. Dudhia J. Troeberg L. Itoh Y. (2017). Inhibition of Shedding of Low‐Density Lipoprotein Receptor-Related Protein 1 Reverses Cartilage Matrix Degradation in Osteoarthritis. Arthritis Rheumatol. 69, 1246–1256. 10.1002/art.40080 28235248 PMC5449214

[B156] Yamamoto K. Troeberg L. Scilabra S. D. Pelosi M. Murphy C. L. Strickland D. K. (2013). LRP‐1‐mediated Endocytosis Regulates Extracellular Activity of ADAMTS‐5 in Articular Cartilage. FASEB J. 27, 511–521. 10.1096/fj.12-216671 23064555 PMC3545526

[B157] Yang C.-Y. Chanalaris A. Troeberg L. (2017). ADAMTS and ADAM Metalloproteinases in Osteoarthritis - Looking beyond the ‘usual Suspects'. Osteoarthritis Cartilage 25, 1000–1009. 10.1016/j.joca.2017.02.791 28216310 PMC5473942

[B158] Yiotakis A. Dive V. (2008). Synthetic Active Site-Directed Inhibitors of Metzincins: Achievement and Perspectives. Mol. Aspects Med. 29, 329–338. 10.1016/j.mam.2008.06.001 18657570

[B159] Zeng W. Corcoran C. Collins-Racie L. A. Lavallie E. R. Morris E. A. Flannery C. R. (2006). Glycosaminoglycan-binding Properties and Aggrecanase Activities of Truncated ADAMTSs: Comparative Analyses with ADAMTS-5, -9, -16 and -18. Biochim. Biophys. Acta (Bba) - Gen. Subjects 1760, 517–524. 10.1016/j.bbagen.2006.01.013 16507336

[B160] Zhang Y. Lai B. S. Juhas M. (2019). Recent Advances in Aptamer Discovery and Applications. Molecules 24. 10.3390/molecules24050941 PMC6429292 30866536

[B161] Zhang Y. Wei X. Browning S. Scuderi G. Hanna L. S. Wei L. (2017). Targeted Designed Variants of Alpha-2-Macroglobulin (A2M) Attenuate Cartilage Degeneration in a Rat Model of Osteoarthritis Induced by Anterior Cruciate Ligament Transection. Arthritis Res. Ther. 19, 175. 10.1186/s13075-017-1363-4 28743292 PMC5526282

[B162] Zhao C.-Q. Zhang Y.-H. Jiang S.-D. Li H. Jiang L.-S. Dai L.-Y. (2011). ADAMTS-5 and Intervertebral Disc Degeneration: the Results of Tissue Immunohistochemistry and *In Vitro* Cell Culture. J. Orthop. Res. 29, 718–725. 10.1002/jor.21285 21437951

[B163] Zhao L. Huang J. Fan Y. Li J. You T. He S. (2019). Exploration of CRISPR/Cas9-based Gene Editing as Therapy for Osteoarthritis. Ann. Rheum. Dis. 78, 676–682. 10.1136/annrheumdis-2018-214724 30842121 PMC6621547

